# 3D‐Printed Functional Hydrogel by DNA‐Induced Biomineralization for Accelerated Diabetic Wound Healing

**DOI:** 10.1002/advs.202300816

**Published:** 2023-04-19

**Authors:** Nahyun Kim, Hyun Lee, Ginam Han, Minho Kang, Sinwoo Park, Dong Eung Kim, Minyoung Lee, Moon‐Jo Kim, Yuhyun Na, SeKwon Oh, Seo‐Jun Bang, Tae‐Sik Jang, Hyoun‐Ee Kim, Jungwon Park, Su Ryon Shin, Hyun‐Do Jung

**Affiliations:** ^1^ Department of Biomedical‐Chemical Engineering The Catholic University of Korea Bucheon 14662 Republic of Korea; ^2^ Department of Biotechnology The Catholic University of Korea Bucheon 14662 Republic of Korea; ^3^ Research Institute of Advanced Manufacturing & Materials Technology Korea Institute of Industrial Technology Incheon 21999 Republic of Korea; ^4^ School of Chemical and Biological Engineering and Institute of Chemical Processes (ICP) Seoul National University Seoul 08826 Republic of Korea; ^5^ Center for Nanoparticle Research Institute of Basic Science (IBS) Seoul 08826 Republic of Korea; ^6^ Department of Materials Science and Engineering Chosun University Gwangju 61452 Republic of Korea; ^7^ Department of Materials Science and Engineering Seoul National University Seoul 08826 Republic of Korea; ^8^ Division of Engineering in Medicine Department of Medicine Harvard Medical School and Brigham and Women's Hospital Cambridge MA 02139 USA

**Keywords:** AI‐based 3D printing, bioinspired hydrogel wound dressing, biomineralization, biosilica, DNA

## Abstract

Chronic wounds in diabetic patients are challenging because their prolonged inflammation makes healing difficult, thus burdening patients, society, and health care systems. Customized dressing materials are needed to effectively treat such wounds that vary in shape and depth. The continuous development of 3D‐printing technology along with artificial intelligence has increased the precision, versatility, and compatibility of various materials, thus providing the considerable potential to meet the abovementioned needs. Herein, functional 3D‐printing inks comprising DNA from salmon sperm and DNA‐induced biosilica inspired by marine sponges, are developed for the machine learning‐based 3D‐printing of wound dressings. The DNA and biomineralized silica are incorporated into hydrogel inks in a fast, facile manner. The 3D‐printed wound dressing thus generates provided appropriate porosity, characterized by effective exudate and blood absorption at wound sites, and mechanical tunability indicated by good shape fidelity and printability during optimized 3D printing. Moreover, the DNA and biomineralized silica act as nanotherapeutics, enhancing the biological activity of the dressings in terms of reactive oxygen species scavenging, angiogenesis, and anti‐inflammation activity, thereby accelerating acute and diabetic wound healing. These bioinspired 3D‐printed hydrogels produce using a DNA‐induced biomineralization strategy are an excellent functional platform for clinical applications in acute and chronic wound repair.

## Graphical abstract

The healing of chronic wounds in diabetic patients is challenging due to prolonged inflammation. In this study, bioinspired 3D printing inks comprising of functionalized sodium alginate (FSA), biomineralized silica, and DNA are developed. Bioinspired hydrogel dressings fabricated with DNA‐bSi@FSA inks and the 3D printing process are optimized using machine learning. The potential of using biomineralization‐based nanotherapeutics for chronic wound repair is evaluated.

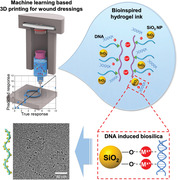

## 1 Introduction

Skin injuries caused by diseases, burns, and other accidents are common wounds.^[^ ^1^ ^]^ Generally, acute wounds undergo the following healing stages: initial hemostasis, inflammation, proliferation, and remodeling.^[^ ^2^, ^3^, ^4^ ^]^ Chronic wounds, in contrast, do not follow these stages; they hardly heal owing to prolonged inflammation and overexpression of reactive oxygen species (ROS), which further exacerbates oxidative stress and reduces angiogenesis, thus leading to abnormal re‐epithelialization and persistent infection and necrosis. Consequently, patients' recovery is delayed and their quality of life ultimately deteriorates.^[^ ^5^, ^6^, ^7^ ^]^ Diabetic wounds are a major chronic complication of diabetes mellitus, and they increase the likelihood of clinical infection and disability and can even cause death.^[^ ^7^, ^8^, ^9^, ^10^, ^11^ ^]^ In 2019, approximately 463 million adults had diabetes across the world; this figure is estimated to reach 578 million by 2030.^[^ ^12^, ^13^ ^]^ It is therefore essential to develop wound dressings that not only function as physical barriers to prevent further damage and infection but also accelerate wound closure to facilitate wound healing and reduce scar formation.^[^ ^14^ ^]^ Numerous dressing materials, such as gauze and cotton, are traditionally used in clinics; however, they lack biological functions and cannot adapt well to irregular wounds.^[^ ^15^ ^]^ Hydrogels are preferable dressing materials because of their excellent biocompatibility, degradation, and natural drug‐loading ability.^[^ ^16^, ^17^ ^]^ In particular, they can also cover irregularly shaped wounds and exhibit excellent moisture retention, which prevents reinjury when dressing is changed.^[^ ^18^, ^19^ ^]^

Sodium alginate (SA) is a marine‐derived polysaccharide that comprises (1,4)‐linked *β*‐D‐mannuronic acid (M) and *α*‐L‐guluronic acid (G) units and is commonly used as a tissue regeneration and drug delivery material. Its architecture is similar to an extracellular matrix (ECM), and it has excellent biocompatibility, low cytotoxicity, high aqueous solubility, and is cost effective.^[^ ^20^, ^21^, ^22^, ^23^ ^]^ Alginate hydrogels can be easily crosslinked with divalent cations, such as Ca^2+^, Ba^2+^, Mn^2+^, and Cu^2+^, to form egg‐box junctions and induce gelation.^[^ ^24^, ^25^ ^]^ Ionic crosslinking occurs rapidly in such hydrogels, thus enabling its use in extrusion‐based 3D printing for medical applications needing patient customization, such as wound dressings, artificial cartilages, biosensors, and drug delivery materials.^[^ ^26^, ^27^, ^28^, ^29^, ^30^ ^]^ However, certain issues hinder the use of alginate in 3D printing applications, such as shape fidelity and structural collapse, which can result in printing failure or geometric inaccuracies.^[^ ^31^ ^]^ Furthermore, alginate hydrogels have low cell proliferation, insufficient cell adhesion, poor mechanical properties, and rapid dissolution rate.^[^ ^32^ ^]^ Nonetheless, alginate hydrogels are being progressively developed and subjected to chemical and physical enhancements and modifications, such as combining with various organic and inorganic therapeutic agents.^[^ ^29^, ^33^, ^34^ ^]^ Polydeoxyribonucleotide (PDRN) is an emerging natural bioactive therapeutic and a deoxyribonucleic acid (DNA)‐derived agent that plays an important role in diverse biological activities, including reduction of inflammation, tissue regeneration, angiogenesis, and cell activity promotion.^[^ ^35^, ^36^ ^]^ PDRN has a molecular weight of 50–1500 kDa and is extracted from *Oncorhynchus keta* (chum salmon) or *Oncorhynchus mykiss* (salmon trout) sperm cells.^[^ ^37^ ^]^ DNA from salmon sperm acts as an adenosine A_2A_ receptor agonist, which can regulate myocardial blood flow, suppress immune cell activation, and modulate tubuloglomerular feedback and renal macrophage number and phenotype.^[^ ^38^ ^]^ DNA from salmon sperm acts as genetic building blocks, nucleotides, and nucleosides, which can help damaged or hypoxic tissues produce nucleic acids efficiently through salvage pathways.^[^ ^39^ ^]^ Moreover, DNA reduces ROS levels and exerts antioxidant activities.^[^ ^40^, ^41^ ^]^ Diverse in vitro, in vivo, and clinical studies suggest that tissue regeneration can be accelerated using DNA with other biomaterials.^[^ ^42^, ^43^, ^44^, ^45^ ^]^

Silica (silicon oxide [SiO_2_]) nanoparticles (NPs), which are representative metal oxide‐based nanotherapeutics, exhibit drug‐loading and osteogenic abilities, biocompatibility, and angiogenesis promotion.^[^ ^14^, ^46^, ^47^, ^48^, ^49^, ^50^ ^]^ In skin tissue, silica NPs can release silicon ions that aid in angiogenesis and collagen deposition during the healing of diabetic wounds by activating the hypoxia‐inducible factor (HIF)‐1*α* and vascular endothelial growth factor (VEGF) signaling pathways.^[^ ^51^, ^52^, ^53^, ^54^, ^55^ ^]^ Moreover, silica NPs exert nanobridging effects on soft tissue matrices that accelerate wound closure.^[^ ^56^, ^57^ ^]^ The biologically beneficial silica is abundantly present in soil and in many unicellular and multicellular living organisms.^[^ ^58^ ^]^ Living organisms such as marine sponges and diatoms can produce hierarchical nanostructured silica architectures through the biomineralization of silica using bottom‐up biomolecular self‐assembly; this biomineralized silica is key to attaining the functional and mechanical properties of organic–inorganic hybridized living organisms.^[^ ^59^ ^]^ DNA, the hereditary molecule in humans and almost all other organisms, is an attractive biomolecule comprising two linked strands in a double‐helix structure. Because of their distinguished hierarchical self‐assembled structure, these helical nanostructures can be structurally and functionally relied on to guide silica biomineralization as the interactions between DNA and the silica precursor are induced. However, DNA cannot induce the formation of silica biominerals because the surfaces of both DNA and silica are highly negatively charged and repel each other; therefore, positively charged molecules, such as cationic surfactants, amphiphiles, and chaotropic agents, are used as cation bridges that directly facilitate the interaction between DNA and biomineralized silica.^[^ ^60^, ^61^ ^]^ Therefore, when using a combination of PDRN and alginate hydrogel, the conjugated DNA‐derived PDRN and biomineralized silica NPs should not only modulate the physical properties but also improve the biological properties of alginate hydrogel as a dressing material. This provides a positive viewpoint on potential therapeutic wound dressing materials for healing chronic wounds in diabetic patients.

In this work, we designed a 3D‐printable material with versatile nature‐derived bioactive hydrogels incorporated with nanotherapeutics to fabricate customized wound dressings for acute and chronic wound treatment. An intrinsically bioactive wound dressing strategy based on biomineralized silica NPs and natural DNA from salmon sperm embedded in functionalized alginate (FSA) hydrogel was developed. Having the advantages of natural organic biomaterials, these nanocomposite hydrogels are expected to consume hydroxyls, scavenge ROS, and promote angiogenesis. Furthermore, the conjugated silica NPs in the hydrogels can synergistically enhance impaired tissue regeneration efficiency while reducing inflammation risks, thus exhibiting considerable potential for use in complete wound‐healing therapies. The physicochemical properties and biological behaviors of the 3D‐printed bioactive hydrogel dressings, including their in vitro biocompatibility and in vivo wound healing ability, are evaluated and discussed in detail.

## 2 Results and Discussion

### 2.1 Synthesis of Bioactive Hydrogel Ink and Preparation of 3D‐Printed Hydrogel Dressing

**Scheme 1:**
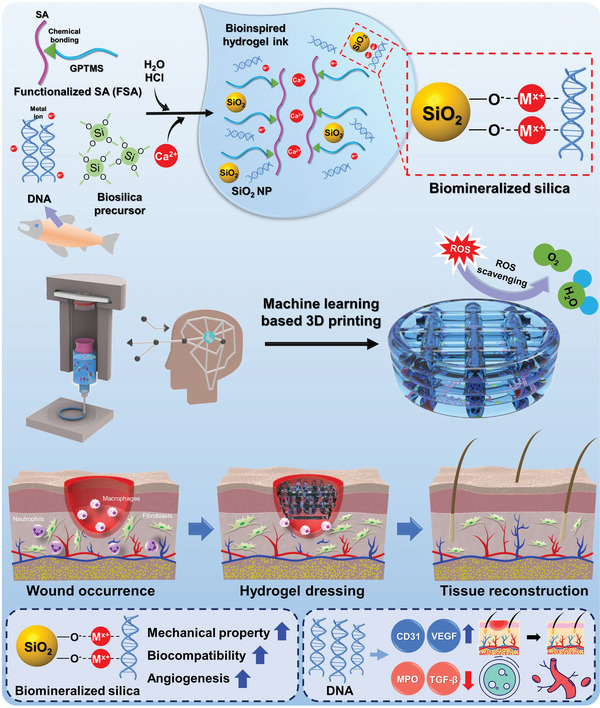
Schematic diagram of the fabrication of bioinspired 3D‐printed hydrogels using DNA‐induced biomineralization.

Recently, alginate‐based hydrogels have been modified using organic polymers and/or inorganic reinforcements. Herein, we designed an enhanced alginate hydrogel ink for wound healing and fabricated bioactive hydrogels incorporated with both organic and inorganic therapeutic agents. The design concept of the 3D‐printed wound dressing and its synthesis process are presented in **Scheme** **1**, including the preparation of the hydrogel ink, machine learning‐based optimized 3D printing, ROS‐scavenging effect, the expected healing process, and the effects of the materials. Our approach of synthesizing bioactive hydrogel ink for diabetic wound healing was based on the excellent mechanical stability, printability, biocompatibility, and ROS scavenging effect of the incorporated materials. The epoxy ring of 3‐glycidoxypropyl trimethoxysilane (GPTMS) was opened by the nucleophilic attack of the carboxylic groups of alginate. GPTMS, a coupling agent, was added to the alginate hydrogel to induce covalent coupling between alginate and silica based on an in situ sol‐gel process.^[^ ^62^ ^]^ DNA was added to the FSA hydrogel as an organic therapeutic agent in the form of a fine aqueous dispersion, and the silica precursor sol was added to produce the bioactive hydrogel ink.

DNA extracted from salmon sperm, also known as PDRN, is a nanotherapeutic agent that has a length of 50–2000 base pairs. It comprises a low‐molecular‐weight DNA with a double‐helix structure with coupled deoxyribonucleotide polymeric chains with purine and pyrimidine nucleotides bonded by phosphodiester bonds. Shin et al. studied the PDRN structure and morphology using atomic force microscopy (AFM), which can image single molecules and protein structures.^[^ ^36^ ^]^ However, it only allows 2D surface analysis; moreover, the AFM tip can easily deform or damage the delicate biological specimens.^[^ ^63^, ^64^ ^]^ Thus, additional analysis was performed using cryogenic transmission electron microscopy (cryo‐TEM) to confirm the 3D volume data. To the best of our knowledge, this is the first study to analyze and report the structure and morphology of PDRN using cryo‐TEM (Figure S1, Supporting Information). The general structure of a short DNA strand with a length of >100 nm and diameter of <10 nm can be clearly observed using cryo‐TEM. There were no agglomerations in the DNA solution, but the strands were randomly and well entangled. The DNA size was measured using dynamic light scattering (DLS). A multimodal hydrodynamic size distribution was found in the DNA solution, with the first, second, and third peaks in the ranges of 0.8–1.1, 2–6, and 200–350 nm, respectively (Figure S2A, Supporting Information). Although commonly used on spherical colloids, DLS analysis can result in a high polydispersity index when used on irregularly shaped samples.^[^ ^65^ ^]^ However, in this study, the long DNA structure led to a multimodal size distribution, and the results were consistent with those of previous AFM analysis showing that PDRN had a thread‐like morphology with a diameter of 0.5–2.5 nm and a length of 100–1200 nm.^[^ ^36^ ^]^ The average surface zeta potential of DNA in this study was −32.0 ± 5.6 mV at pH 10.5 (Figure S2B, Supporting Information); this is consistent with the results of previous studies, indicating that the natural DNA was negatively charged and had sufficient dispersion and colloidal stability.^[^ ^35^, ^66^ ^]^ We postulated from the DNA structure and morphology obtained from cryo‐TEM and DLS that its morphology was a long, thin chain.

Both silica surfaces and DNA are highly negatively charged owing to the presence of silanol groups. Thus, strong electrostatic repulsion occurs between DNA and silica surfaces. Cation bridges can be used to connect the backbone of DNA and the silanol groups of the silica surface to overcome their electrostatic repulsion. Generally, extracted DNA contains inorganic salts, which form a uniform solution and prevent phase separation. In this study, the DNA from salmon sperm included sodium salt.^[^ ^67^ ^]^ Monovalent cations, such as Na^+^, weakly form ionic or electrostatic forces with the negatively charged phosphates of the DNA backbone, whereas divalent cations can form indirect and/or covalent bonds with the phosphates or silanols on silica.^[^ ^68^, ^69^, ^70^ ^]^ Here, we hypothesized that 1) the sodium salt in DNA could be primarily used as a monovalent cation bridge to connect with the negatively charged silica and 2) Ca^2+^ from calcium chloride (CaCl_2_) as a hydrogel crosslinker would facilitate a strong binding between the phosphate backbone of DNA and silica, which will act as divalent cation. Another hypothesis was that the pH can be modified to suppress the electrostatic repulsion between DNA and silica as silica surfaces show neutral or slightly positive charges below their isoelectric point, which is approximately at pH 2.^[^ ^71^ ^]^ The gelation time of silica precursors, such as tetramethoxysilane (TMOS) and tetraethoxysilane (TEOS), depends on the pH environment; their hydrolysis rate is relatively higher in acidic environments, whereas their condensation rate is faster in basic environments, leading to rapid gelation.^[^ ^72^ ^]^ Therefore, the silica precursor at low pH levels in this study should have the following advantages: 1) It should easily connect with the negatively charged phosphate groups of DNA by diminishing the negatively charged surface. 2) It should facilitate fast gelation and biomineralization upon encountering alkaline DNA@FSA ink. In accordance with this complex strategy, gelated silica after the incorporation of DNA@FSA ink might appear as silica NPs and interact with DNA (through electrostatic bonding) and functionalized alginate (through covalent bonding).

**Figure 1:**
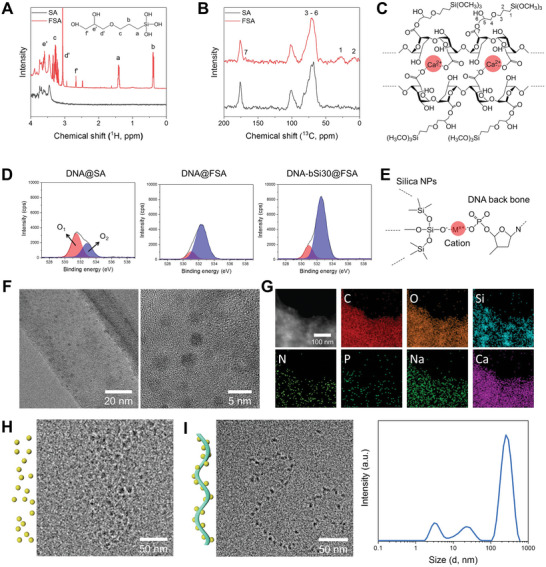
Results of A) liquid‐state ^1^H NMR and B) solid‐state ^13^C NMR of the pristine alginate and prepared functionalized alginate (FSA) hydrogel. C) Chemical structure of the prepared hydrogel inks comprising ionically crosslinked FSA based on NMR results. D) XPS results (O 1s) of DNA@SA, DNA@FSA, and DNA‐bSi30@FSA, and E) schematic diagram of DNA‐conjugated silica NPs. F) Representative TEM images of synthesized silica NPs in DNA@FSA ink at different magnifications. G) STEM and elemental mapping images of DNA@FSA ink. H) Silica NPs present in Si@FSA ink observed with cryo‐TEM. I) Biomineralized silica NPs on DNA with a ribbon‐like structure present in DNA‐bSi@FSA inks and their size distribution.

For further verification, liquid‐state ^1^H and solid‐state ^13^C NMR analyses were conducted to confirm the formation of an additional bond between the alginate and GPTMS using a previously reported protocol.^[^ ^62^ ^]^ First, methanol‐related peaks appeared after hydrolysis of the methoxysilane group of GPTMS under acidic conditions, and an f’ peak appeared, indicating the epoxide opening of GPTMS as illustrated in the liquid‐state ^1^H NMR analysis (**Figure** **1**A). Moreover, a peak related to the carboxylic ester group, which is noted as the seventh peak in Figure 1B, appeared; this peak was observed for the pure alginate, suggesting that epoxide‐opened GPTMS was covalently bound to the alginate. Based on these results, we determined that the chemical structure of the fabricated FSA comprised an egg box‐like structure formed by the ionically crosslinked alginate with GPTMS functionalization, as schematically presented in Figure 1C. XPS analysis was performed on DNA@SA, DNA@FSA, and DNA‐bSi30@FSA to investigate the mechanism underlying the interaction between DNA and silica NPs (Figure 1D and Figure S3, Supporting Information). As shown in Figure 1D, the intensity of the O_1_ peak, corresponding to the C=O, P=O, and P—O^−^ groups, was greater than that of the O_2_ peak, which corresponds to the C—O—C and C—O—P groups in DNA@SA. However, this trend was inverted for DNA@FSA and DNA‐bSi30@FSA. This shift could be derived from the electrostatic attraction of positively charged components. Considering that the cationic molecules are electrostatically attracted to negatively charged silica,^[^ ^60^, ^73^ ^]^ the generated silica NPs could bind to the DNA backbone through the abundant cationic bridges provided by Ca^2+^ and Na^+^ (Figure 1E).

We synthesized a silica precursor with a positively charged surface by adding hydrochloric acid and distilled water (DW) into TMOS. The silica precursor had a slightly positive surface zeta potential of 1.12 ± 0.74 mV at pH 1.6 (Figure S4A, Supporting Information). Spherical silica NPs with a diameter of approximately 3 nm were observed on dry‐state TEM imaging of the synthesized bioactive hydrogel inks (Figure 1F). Furthermore, TEM imaging revealed that homogeneous silica NPs were dispersed in the hydrogel inks. Scanning transmission electron microscopy (STEM) images and energy‐dispersive X‐ray spectroscopy (EDS) elemental mapping analyses showed that in addition to the Si and O signals from the silica, C, N, P, Na, and Ca signal traces in DNA@FSA ink were homogeneously dispersed throughout the bioactive hydrogel inks, suggesting that the silica NPs were well dispersed in the pre‐crosslinked bioactive hydrogel inks (Figure 1G). However, dry‐state TEM analysis did not indicate any evidence of biomineralized silica on DNA. Thus, cryo‐TEM was used to determine and visualize the binding structure of the DNA and biomineralized silica. The Si@FSA samples were assessed using cryo‐TEM imaging (Figure 1H) to compare their silica NP sizes and arrangements. Monodispersed silica nanospheres sized 5–10 nm were uniformly observed in the Si@FSA ink without agglomeration, indicating their sufficient dispersion and colloidal stability due to the covalent coupling ability of GPTMS. In contrast, the DNA‐bSi@FSA inks contained ribbon‐like strand structures with silica NPs along their surfaces (Figure 1I). Similar structures have been reported in other studies on self‐assembled organism–inorganic NP superstructures.^[^ ^74^, ^75^, ^76^, ^77^ ^]^ In terms of the size of DNA and silica NPs, a previous study stated that the monodispersity of silica, which is spatially ordered, along DNA is inevitable; this ordered NP distribution is attributed to the minimization of electrostatic repulsion between neighboring NP–organism amphiphile aggregates.^[^ ^75^ ^]^ The size‐distribution trend of the DNA‐bSi@FSA inks was similar to that of the DNA solution; however, its graph was slightly shifted to the right, with the first, second, and third peaks in the range of 2–7, 10–50, and 100–600 nm, respectively, owing to the biomineralized silica NPs on the DNA (Figure 1I). As shown in Figure S4B (Supporting Information), the surface zeta potential of the DNA‐bSi@FSA inks was −80.1 ± 1.9 mV at pH 7.9, indicating that the basic DNA@FSA ink was neutralized by the silica sol. The synthesized bioactive hydrogel inks were highly negatively charged because all hydrogel components, including alginate, had high negative charge densities.^[^ ^78^ ^]^ Therefore, the sol‐gel‐based silica precursor was successfully biomineralized onto the DNA and dispersed in the bioactive hydrogel inks. The DNA‐based organisms interacted with the inorganic species source and biomineralized and assembled them into ordered structures. DNA‐based biomimetics can induce rapid silica gelation from an Si source and trigger the biomineralization of silica‐like marine organisms.

**Figure 2:**
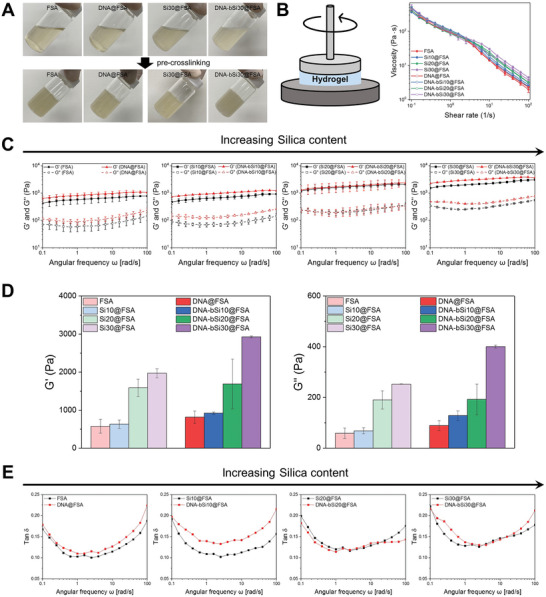
A) Optical images of the prepared bioactive hydrogel inks with flowability and pre‐crosslinked inks exhibiting solid‐like behavior. B) Schematic diagram of measured viscosity of bioactive hydrogel inks and monitored viscosity in terms of shear rate. C) Storage modulus (*G*′) and loss modulus (*G*″) of bioactive hydrogel inks with different silica concentrations based on DNA presence. D) Quantified G′ and G″ at an angular frequency of 1 Hz. E) Calculated tan *δ* of bioactive hydrogel inks.

Alginate hydrogels exhibit non‐Newtonian behaviors and possess printable rheological properties suitable for extrusion‐based 3D printer nozzles.^[^ ^79^ ^]^ However, they have inadequate viscoelasticity; for instance, their viscosity recovery (rigidity) is insufficient for maintaining the printed architecture.^[^ ^33^ ^]^ Thus, alginate hydrogels require rheological enhancement to attain viscoelastic properties suitable for extrusion‐based printing and withstand limitations, such as 3D printing failure and geometric inaccuracies. Before quantitative analyses were conducted, a markedly reduced flowability of the hydrogel inks was observed after precrosslinking using a CaCl_2_ solution, proving its structural sustainability (**Figure** **2**A). The rheological properties of the bioactive hydrogels were characterized to verify their ability to sustain the architecture and withstand the stress applied during extrusion with the incorporation of DNA and silica. As shown by the flow viscosity curves at shear rates of 0.1–100 s^−1^ (Figure 2B), the viscosity of the bioactive hydrogel inks decreased by more than two orders of magnitude as the shear rate increased, demonstrating shear thinning across a range of concentrations, which is a typical behavior of non‐Newtonian fluids.^[^ ^80^ ^]^ With a larger amount of DNA added and an increase in silica content, the viscosity of the hydrogel inks increased, demonstrating that the physiological interaction between the nanotherapeutics and FSA could improve the viscosity of the hydrogel inks with biomineralized nanotherapeutics. The shear thinning enabled the stable extrusion of the inks without any agglomeration of the silica or DNA nanotherapeutics and protected the laden cells from shear force and pressure in the nozzle. As shown in Figure 2C, the rheological properties of the bioactive hydrogels were evaluated using a rheometer in angular frequency sweep mode at a fixed strain. The two dynamic moduli generally increased as the frequency increased from 0.1 rad s^−1^ to 100 rad s^−1^, reaching their maximum values at 100 rad s^−1^. The storage moduli (*G*′) of all hydrogels were approximately nine times larger than their loss moduli (*G*″), suggesting that they generally had elastomeric characteristics based on polysaccharides and behaved like soft solid gels during 3D printing.^[^ ^80^ ^]^ The increases in the *G*′ and *G*″ values tended to increase with the silica concentration of the hydrogel inks, indicating a physically strong crosslinked network of hydrogel inks within the silica. With the addition of DNA to the hydrogel inks, the *G*′ and *G*″ values of the hydrogel inks with DNA increased further. This phenomenon was remarkable at high silica concentrations; the hydrogel ink containing 30 wt% silica and DNA (DNA‐bSi30@FSA) showed a larger increase in the *G*′ value compared with the DNA containing FSA inks. With an increase in the silica content from 0 wt% to 30 wt%, the *G*′ value of DNA@FSA with biomineralized silica was greatly enhanced from approximately 1970 to 2930 Pa, and that of DNA@FSA without silica increased from approximately 670 to 965 Pa at 1 Hz (Figure 2D). Hydrogel inks for 3D printing need sufficient interconnections between layers to maintain the integrity of printed architectures, so the interaction between their solid and liquid phases should be considered. The influence of the loss tangent (tan *δ*, calculated as *G*″/*G*′) on angular frequency, which is directly related to the strength in maintaining the fabricated architecture, was investigated to understand the viscoelastic nature of the fabricated bioactive hydrogel inks. As seen in Figure 2E, the tan *δ* values of all hydrogel inks initially decreased with an increase in angular frequency, passed a distinct minimum, and increased again. The closer the tan *δ* value to 0, the more solid‐like the elastic behavior; in contrast, the closer it is to 1, the more liquid‐like the elastic behavior.^[^ ^79^ ^]^ *G*′ and *G*″ were assessed as functions of the angular frequency (Figure 2C). The *G*′ values of all hydrogels were higher than their *G*″ values; thus, the tan *δ* values were <1, which meant that these hydrogel inks could maintain elasticity at shear rates of 0.1–100 rad s^−1^. However, the tan *δ* values increased slightly when high‐concentration silica and DNA were added to the hydrogel inks, indicating the formation of a stronger 3D network of crosslinks in the bioactive hydrogel inks. Gao et al. showed that gelatin–alginate composite hydrogel inks with tan *δ* values of 0.25–0.45 had sufficient smoothness and structural integrity.^[^ ^81^ ^]^ Although the tan *δ* value of the DNA‐bSi30@FSA ink, which was the highest among all hydrogel inks, was not ideal, a sufficient tan *δ* (low *G*′ and *G*″) was obtained for 3D printing (Figure 2E). Hence, the fabricated hydrogel inks could maintain their elasticity at the applied shear rates, indicating the strong mechanical stability of the nanocomposite hydrogel.

3D printing technology using diverse biomaterials has garnered increasing attention in tissue engineering applications because of its benefits, such as creating complex shape, patient customization, and cost‐ and time‐effectiveness.^[^ ^82^, ^83^, ^84^, ^85^ ^]^ Although the hydrogel‐based 3D printing process is developing radically, deciding on printing strategies for newly developed hydrogel inks remains challenging. Herein, we preliminarily designed a 3D‐printable hydrogel ink using a machine learning‐based model system comprising functionalized alginate and DNA (DNA@FSA inks). A pneumatic 3D bioprinter was used to create a mesh‐type dressing model (10 × 10 × 2 mm^3^). The nozzle size (0.2 and 0.4 mm), printing temperature (27 °C and 37 °C), pneumatic pressure (20 and 60 kPa), and gel concentration (1, 2, 2.5, 3, and 4 w/v%) were adjusted independently. The feed rate of the nozzle was kept constant as 3 mm/min for all experiments. After the completion of the 3D printing, the printability score of each sample was evaluated based on (i) whether the strand was printed or not (good printing: 2, fair printing: 1, poor printing: 0) and the degree of similarity of the printed sample to the target design regarding the (ii) horizontal and vertical lengths (good printing: 4, fair printing: 2, poor printing: 0) and (iii) height (excellent printing: 6, good printing: 4, fair printing: 2, poor printing: 0). The results of the printability assessment are described in detail in Table S1 (Supporting Information). All of the printed samples were scored according to three criteria, and the total score was summed up to confirm the printability characteristics according to the combination of the test matrix.

**Figure 3:**
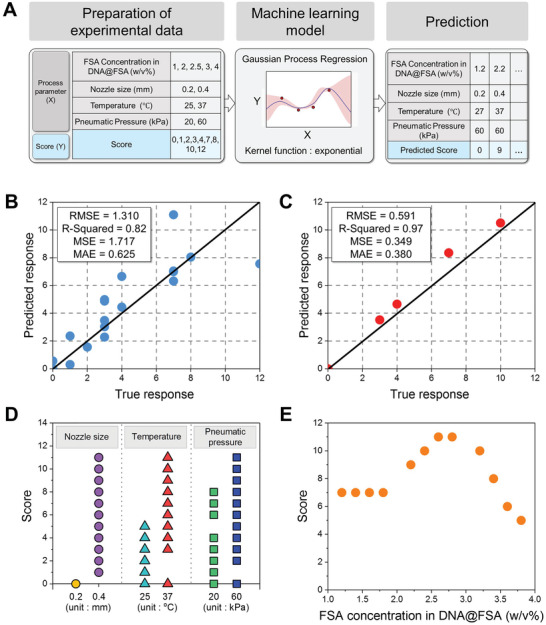
A) Flow chart of machine learning modeling using Gaussian process regression. B) Trained model validation (FSA concentration in DNA@FSA: 1, 2, 3, and 4 w/v%). C) Trained model test (FSA concentration in DNA@FSA: 2.5 w/v%). Score according to variable D) nozzle size, temperature, pneumatic pressure, and E) FSA concentration.

The flow chart of machine learning modeling to predict the printability score using the newly developed hydrogel inks is presented in **Figure** **3**A. The dataset used for machine learning modeling comprised 40 (2 × 2 × 2 × 5) datapoints. Each datapoint was composed of four input variables, corresponding to nozzle size, printing temperature, pneumatic pressure, and FSA concentration, and one output variable, corresponding to the printability score. The Gaussian process regression (GPR) model with a nonisotropic rational quadratic kernel function was applied to develop the machine learning model. The GPR model was adopted because it is particularly useful when working with a small number of data sets, in which case the model may be prone to overfitting, and uncertainty in the predictions can help prevent overconfidence in the model.^[^ ^86^ ^]^ Thus, GPR has been applied widely to the fields of engineering,^[^ ^87^ ^]^ robotics,^[^ ^88^ ^]^ chemical,^[^ ^89^ ^]^ and biology.^[^ ^90^, ^91^, ^92^ ^]^ In addition, the rationality of the GPR model in this research was confirmed by comparing the confidence interval and the evaluated scores. As demonstrated in Figure S5 (Supporting Information), all evaluated scores were within the 95% confidence interval, the size of which was relatively small. The model was trained with the dataset using FSA concentrations of 1, 2, 3, and 4 w/v%, then tested with the dataset using an FSA concentration of 2.5 w/v%. The accuracy (*R* ^2^) of the trained model for validation and testing was 82% and 97%, respectively, as shown in Figure 3B,C. This result indicated that the algorithm found a relatively good fit for the trained model test in the course of the training. We were interested in deriving a variable level of input, to obtain the best quality of printing at narrower FSA concentration intervals. Using the machine learning model developed here, the printability scores for a total of 96 (2 × 2 × 2 × 12) datasets were predicted by combining the input variables of nozzle size (0.2 and 0.4 mm), printing temperature (27 °C and 37 °C), pneumatic pressure (20 and 60 kPa), and FSA concentration (1.2, 1.4, 1.6, 1.8, 2.2, 2.4, 2.6, 2.8, 3.2, 3.4, 3.6, and 3.8 w/v%). To evaluate the effect of each input variable on printability, the scores were sorted according to each level of the input variable, as shown in Figure 3D. Higher scores were observed under the conditions of a nozzle size of 0.4 mm, a printing temperature of 37 °C, and a pneumatic pressure of 60 kPa. It is worth noting that a higher score was obtained when the printing temperature was 37 °C, which is the optimized condition for cell‐laden bioprinting. Using these selected levels of the input variables, the predicted scores were plotted against the FSA concentration (Figure 3E). It was observed that the printability score increased to up to 3 w/v% of the FSA concentration and decreased when the gel concentration increased to 3 w/v% or more. The highest printability score was predicted at FSA concentrations of 2.6 and 2.8 w/v%. Therefore, based on the machine learning methodology described in this study, an FSA concentration of 3 w/v% was used to stabilize and optimize the printing process.

### 2.2 Physicochemical Characterization of 3D‐Printed Hydrogel Dressing

**Figure 4:**
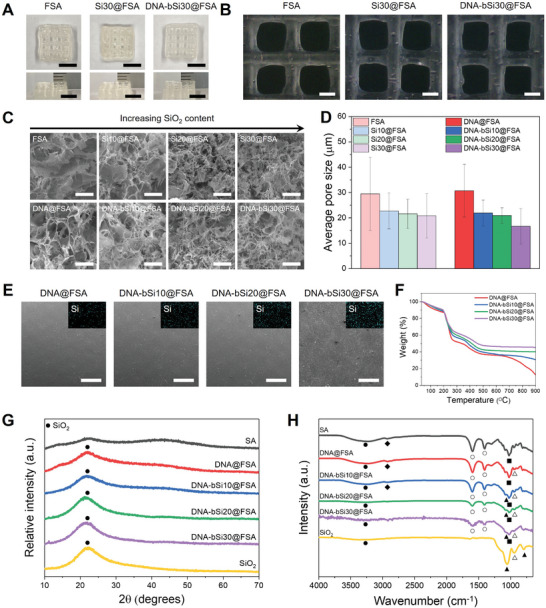
A) Optical images of 3D‐printed hydrogel dressings captured from top and side perspectives (scale bar: 5 mm). B) Demonstration of the hydrogel dressings with a lattice structure using optical microscopy (scale bar: 1 mm). C) 3D microstructure of freeze‐dried hydrogel dressings composed of micropores (scale bar: 50 µm). D) Average pore size under each condition (*n* = 10). E) Typical FE‐SEM images and Si distributions of air‐dried DNA‐incorporated hydrogel scaffolds (scale bar: 500 µm). F) Monitored weight decrease from TGA. G) XRD patterns and (H) FT‐IR spectra of the alginate, silica, and DNA‐Si@FSA nanocomposites (●: —OH group, ♦: C—H Stretching, ○: Vibrations of asymmetric elongation of the C—O bond of the COO group, ▲: Si—O—Si bond, ■: antisymmetric elongation of C—O—C, and △: Si—OH bond).

According to Figure S6 (Supporting Information), as the silica concentration in the hydrogel inks increased from 0 wt% to 30 wt%, the transparent clear inks became transparent yellow and exhibited homogeneous distribution, indicating uniform silica dispersion. All hydrogel inks presented in the sol state and began undergoing sol‐gel transition within minutes of being mixed with the CaCl_2_ solution. A 3D printing test was conducted on samples of the designed dressings sized 10 mm × 10 mm × 2 mm to investigate the printability of the synthesized hydrogel inks with various silica concentrations, as shown in **Figure** **4**A. All of the proposed hydrogel inks were successfully printed with good shape fidelity, and the crosslinked hydrogel dressings were self‐supported and had well‐defined boundaries, suggesting the excellent printability of the proposed hydrogel inks. The most notable advantage of hydrogel 3D printing is that it can create an on‐demand customized structure that fits in the wound site.^[^ ^93^, ^94^ ^]^ As an approach to verify the feasibility of the 3D‐printed DNA‐bSi@FSA hydrogel for generating a tailored structure, three different designs (i.e., circular, triangular, and polygonal structures) were printed with non‐lattice and lattice patterns. As shown in Figure S7A (Supporting Information), six types of hydrogel scaffolds were successfully produced with identical dimensions. Complex structures with differed XYZ dimensions were also printed with and without a supporting material, revealing that DNA‐bSi@FSA hydrogel‐based 3D printing could withstand the fabricated structure. The possibility of performing a bench‐to‐clinical‐practice translation is illustrated using a diabetic ulcer foot model (Figure S7B, Supporting Information). When a wound occurs, the wound site would be scanned to prepare an exact 3D model for 3D printing. After obtaining the 3D model, it would be transferred to a printing machine equipped with the DNA‐bSi@FSA hydrogel ink and converted into a toolpath for 3D printing (Video S1, Supporting Information). Subsequently, the printed scaffolds would be crosslinked and applied to the wound site. One of the noticeable merits of this procedure is that it can be performed on‐site in a sterile operating room, to produce a perfectly fitted protective barrier.^[^ ^95^, ^96^, ^97^ ^]^

The surface features of hydrogel wound dressings are important as their 3D structure is directly related to the attachment and growth of tissue cells during wound healing and oxygen exchange.^[^ ^15^, ^18^ ^]^ In addition, the porosity of the wound dressing materials is vital because it critically affects the absorption of exudates and blood.^[^ ^98^, ^99^ ^]^ As shown in Figure 4B, all hydrogels presented with lattice structures with uniformly arranged macropores designed using computer‐aided design (CAD). The internal microstructures of the freeze‐dried hydrogel wound dressings were studied using FE‐SEM to indirectly compare the porous structure. Observation under high‐magnification revealed interconnected 3D microstructures with micropores in all hydrogel dressings, which were designed to facilitate oxygen transport, nutrient transport, and wound waste exchange (Figure 4C).^[^ ^100^ ^]^ The porosities of all hydrogel dressings were >95% and not significantly different. Interestingly, the hydrogel dressings incorporated with silica exhibited a different morphology. With an increase in silica in the FSA, smaller pores were formed between the large original pores, and the pore structure was unimodal. The density of the crosslinked structural network played a major role in determining the pore size of the hydrogels.^[^ ^18^ ^]^ Therefore, the covalent interaction between the silica NPs and FSA increased with the silica NP content, and the pore size of the hydrogel dressings decreased with an increase in the crosslinking density of the hydrogel dressings. Moreover, the structure of the DNA‐bSi@FSA samples was similar to that of marine sponges, which is multiscale and hierarchical in nature, indicating that the biomineralization in DNA‐bSi@FSA mimicked the geometrically complex, siliceous skeletal systems of marine sponges.^[^ ^101^ ^]^ As shown in Figure 4D, the pore sizes of FSA and Si30@FSA were 28.6 and 20.5 µm, respectively, which was not significantly different; the pore sizes of DNA@FSA and DNA‐bSi30@FSA were 29.7 and 16.7 µm, which were significantly different (*p* < 0.001). A higher silica concentration resulted in a denser entanglement of hydrogel networks, thus markedly decreasing the pore sizes of the hydrogels. EDS mapping was performed to study the elemental distribution within the hydrogel dressings (Figure 4E). The Si element originated from GPTMS and silica was homogeneously dispersed in all hydrogels, indicating that the silica was successfully incorporated and uniformly distributed on the nanoscale within the DNA‐bSi@FSA matrices the biomineralization (via DNA) and organic–inorganic covalent coupling (via GPTMS).

The difference in the thermal tolerance of the crosslinked hydrogels was examined via TGA. In the TGA thermogram of FSA, a typical three‐stage thermal degradation process was observed (Figure 4F). In the first stage (30 °C–100 °C), a weight decrease of approximately 10 wt% occurred due to vaporization and dehydration. In the second stage (100 °C–350 °C), approximately 50 wt% alginate was lost, presumably depolymerized by the breakdown of saccharide rings, which are the backbone of the alginates. In the final stage (over 350 °C), approximately 20 wt% weight loss occurred, possibly corresponding to the pyrolysis of the FSA residues. In all bioactive hydrogels with silica, decomposition was almost complete >700 °C, and the fraction of the residue remaining at 900 °C increased from 12.7 wt% for FSA to 45.5 wt% for DNA‐bSi20@FSA, suggesting an agreement between the designed silica concentrations and those obtained from the TGA results.

Figure 4G presents the XRD patterns of the alginate, DNA@FSA samples, silica, and their nanocomposites, to confirm the phase of each hydrogel. No additional impurity peaks were observed in the hydrogels, indicating the high purity of the intended compositions of the hydrogel dressings. The XRD pattern of the crosslinked alginate hydrogel showed two broad diffractions with low intensities at 14.0°, 21.9°, and 40.8°, whereas only one broad peak of silica was observed at 22.0°, indicating that both the alginate and silica had amorphous structures.^[^ ^102^ ^]^ After the alginate was functionalized using GPTMS, the main peak at 2*θ*   =  21.9° sharpened, albeit to a small extent. No peaks other than those of the amorphous alginate hydrogel and silica were observed in the DNA‐bSi@FSA samples. We could not confirm the change in the accurate phase of the bioactive hydrogels because of the overlapping of the main peaks of the amorphous alginate hydrogels with those of the silica NPs. Thus, we assumed that the relative intensities of the alginate peaks detected at 14.0° and 40.8° decreased with the increase in the silica concentration of the hydrogels.

The FT‐IR analysis findings were analyzed to explore the functional bonding of the crosslinked bioactive hydrogel dressings. As shown in Figure 4H, the FT‐IR spectra of the sol‐gel‐based pristine silica showed characteristics typical of silicate networks at 1053 cm^−1^ (asymmetric stretching vibration of Si—O—Si), 945 cm^−1^ (asymmetric bending and stretching vibration of Si—OH), 791 cm^−1^ (symmetric stretching vibration of Si‐O‐Si), and 3359 cm^−1^ (stretching vibration of O—H).^[^ ^103^, ^104^ ^]^ The main bonds of pristine alginate were observed at the following locations: C—O stretching vibration at 1025 cm^−1^; asymmetric stretching vibration of C—O of the COO− group at 1407 and 1595 cm^−1^; C—H stretching vibration at 2930 cm^−1^; O—H stretching vibration at 3224 cm^−1^.^[^ ^36^, ^105^, ^106^ ^]^ In addition, the bands at 949, 885, and 813 cm^−1^ indicated the existence of guluronic and mannuronic acids, respectively in alginate hydrogels.^[^ ^107^ ^]^ Compared with the FT‐IR spectra of the alginate hydrogel and FSA, those of the DNA‐bSi@FSA samples showed relatively stronger adsorption peaks corresponding to the Si—O—Si asymmetric stretching vibration at 1053 cm^−1^, which confirmed that silica was successfully incorporated into FSA. The peaks of FSA, such as those corresponding to C—O stretching vibration, C—H stretching vibration, and guluronic and mannuronic acids, disappeared as the silica concentration of FSA increased.

**Figure 5:**
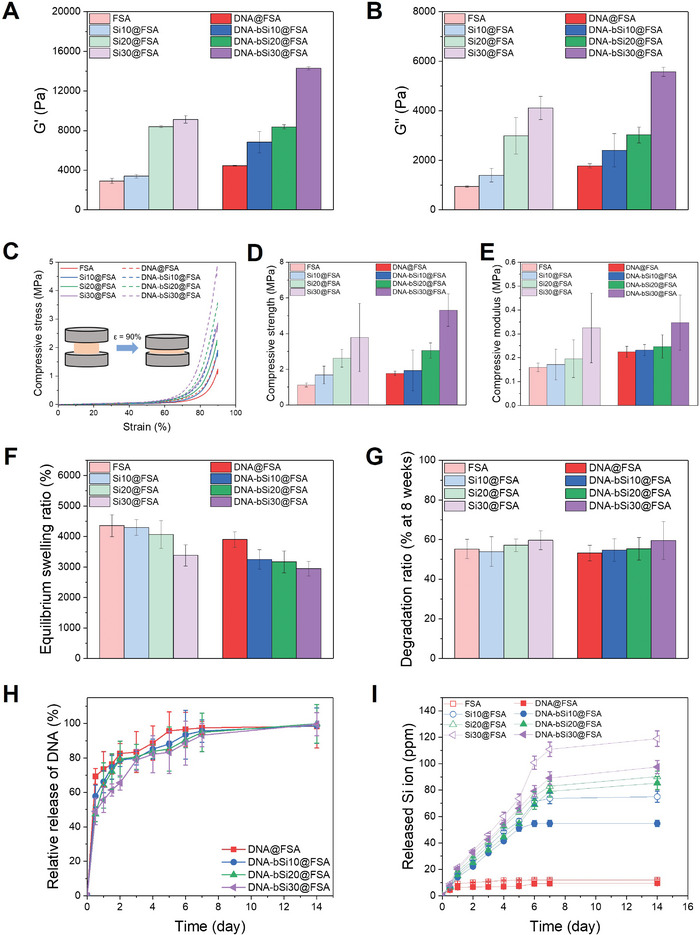
A) *G*′ and B) *G*″ of crosslinked hydrogel dressings at 1 Hz. C) Representative compressive stress–compressive strain (SS) curves of hydrogels up to 90% strain. D) Compressive strength and E) compressive modulus of the hydrogels, from the compression test. F) Equilibrium swelling ratio of hydrogel dressings on day 2 after immersion in DPBS. G) Degradation rates of the prepared hydrogels at week 8 of monitoring. H) Relative DNA release profile of DNA‐incorporated hydrogels and I) Si ion‐release profile of crosslinked hydrogel dressings, as measured using ICP‐MS.

Hydrogels utilized as clinical wound dressings should have good gelling ability, spreading ability, and fluidity to conform to wounds with irregular shapes and varying depths.^[^ ^108^ ^]^ Moreover, they need to protect wound surfaces from the internal and external mechanical deformations that can occur during wound repair.^[^ ^15^ ^]^ Therefore, rheological analysis was performed to compare the deformation resistance of the 3D‐printed hydrogel dressings quantitatively. As seen in **Figure** **5**A and Figure S8A (Supporting Information), *G*′ and *G*″ further increased after crosslinking compared with those of the hydrogel inks, with the G′ value rising by approximately five times when the hydrogel inks were immersed in the 0.3 w/v% CaCl_2_ solution; these findings indicated the gelation of the bioactive hydrogel inks in the CaCl_2_ solution. This was accompanied by a sharp increase in G″, as shown in Figure 5B. After crosslinking, the *G*″ value of the 3D‐printed bioactive hydrogel dressings at 1 Hz rose to approximately 5569 Pa from a pre‐crosslinking value of about 401 Pa. These variations in the *G*′ and *G*″ values induced a dramatic increase in tan *δ*; nonetheless, the tan *δ* remained under 1, suggesting high deformation resistance and an ability to withstand a certain frequency and maintain the gel structure (Figure S8B, Supporting Information).^[^ ^15^ ^]^ The high post‐crosslinking *G*′ value of the 3D‐printed hydrogel dressings showed that the chains were entangled more tightly, suggesting a higher crosslinking density.^[^ ^36^ ^]^ Likewise, *G*′ increased after the inclusion of DNA and silica of increasing concentrations, thereby forming stronger crosslinked networks. The ionically crosslinked alginate networks, which played a key role in dissipating the deformation energy, and the hydrogel dressings incorporated with DNA and biomineralized silica through electrostatic attraction forces could induce additional bonding, thereby enhancing the mechanical properties of the hydrogels.^[^ ^108^, ^109^ ^]^ GPTMS and silica NPs covalently bind during the sol‐gel process, and the epoxy ring of the former was converted into a diol by the nucleophilic attack of alginate.^[^ ^62^, ^110^, ^111^ ^]^ Shin et al. explained that PDRN plays a role in creating semi‐interpenetrating hydrogel networks, which enhance the mechanical properties of hydrogels, although its strength cannot compete with that of other strengthening fillers, such as graphene oxide and nanoclay.^[^ ^99^ ^]^ The well‐distributed DNA observed in the current study exerted nanoscale reinforcement effects that were similar to those described by Shin et al.^[^ ^36^ ^]^ and enhanced the dispersion of stress from the polymer chains to the DNA. This improvement in the physical entanglement degree between the two polymers increased the mechanical sturdiness of the final hydrogels.

A compression test was subsequently performed to further investigate the mechanical properties of the hydrogels, and their stress–strain curves are shown in Figure 5C. All hydrogels possessed a typical profile of ductile, porous polymers under compression, consisting of a linear initial elastic response, extended middle plateau followed by a densification region, and a sharp stress‐drop regime.^[^ ^79^ ^]^ In the final regime, the hydrogels had a strain of approximately 90%. The densification strain was not reduced despite the decrease in the porosity of the hydrogel dressings with the addition of DNA and an increase in silica concentration. A higher silica concentration improved the compressive strength of the printed hydrogel dressings. As shown in Figure 5D, the compressive strength of Si30@FSA (3.79 ± 1.91 MPa) was 3.5 times higher than that of FSA (1.12 ± 0.11 MPa). Hence, the incorporation of well‐dispersed, homogeneous, fine silica NPs enhanced the compressive strength of Si30@FSA. Moreover, the compressive strength of DNA‐bSi30@FSA was 5.32 ± 0.91 MPa, which was around three times higher than that of DNA@FSA, which was 1.78 ± 0.12 MPa, and approximately 4.8 times higher than that of FSA. This significant improvement in compressive strength might have been due to the covalent bonding interaction of the silica NPs and the GPTMS in the alginate chain and the considerable physical entanglement between DNA and FSA, which reduced the hydrogel pore size (Figure 4C, D). The compressive moduli, which reflect the stiffness of the hydrogel dressings, were calculated using the slopes of the linear elastic parts of their stress–strain curves, as shown in Figure 5E. The moduli of all 3D‐printed hydrogel dressings were higher than that of the adjacent subcutaneous tissue, which was about 4.5–8 kPa, suggesting that the alginate‐based hydrogels could fulfill the mechanical specification required for wound dressing applications.^[^ ^15^, ^112^, ^113^, ^114^ ^]^ The compression moduli of the 3D‐printed hydrogel dressings progressively increased with a rise in silica concentration of up to 30 wt%, regardless of the presence of DNA. After the DNA was embedded in the hydrogels, the compressive moduli of the bioactive hydrogel dressings increased from 0.22 ± 0.02 MPa to 0.35 ± 0.12 MPa with an increase in silica concentration; the latter value is closer to that of human skin (0.5–1.95 MPa), ensuring the suitability of these dressings for skin wound therapy.^[^ ^115^, ^116^, ^117^ ^]^ The mechanical properties of hydrogels containing silica are determined by the interaction between the organic and inorganic phases.^[^ ^118^, ^119^ ^]^ In our study, the hydrogel dressings with small amounts of GPTMS could efficiently increase the interaction between the silica NPs and alginate hydrogels without any noticeable aggregation, thereby improving the rheological and compressive properties of the dressings. The biomineralization ability of the DNA induced the formation of biosilica NPs, thus maintaining the mechanical stability of the hydrogel dressings. Moreover, the strengthened storage and compressive moduli confirmed that the DNA nanotherapeutic played a key role in reinforcing the bioactive hydrogel dressings by transferring stress from the alginate hydrogel chains to the DNA. This finding was consistent with the results of the mechanical properties test. Compared with existing alginate‐based wound dressings, the proposed 3D‐printed bioactive hydrogel dressings exhibited higher resistance to external shear and compressive forces. These rheological and compressive results suggested that physical and chemical crosslinking enhanced the mechanical properties of the 3D‐printed bioactive hydrogel dressings. Given their excellent mechanical properties, the 3D‐printed bioactive hydrogel dressings can preserve printed architectures and protect wounds against external forces and additional injury during the healing process.

### 2.3 In Vitro Swelling, Degradation, and Nanotherapeutics Release of 3D‐Printed Hydrogel Dressings

Swelling and water absorption are essential properties of hydrogel dressings for absorbing exudates from chronic wounds, transmitting nutrients and metabolites, and reducing infections for accelerated wound healing.^[^ ^3^, ^120^ ^]^ We evaluated the swelling performance of the hydrogel dressings in Dulbecco's phosphate buffered saline (DPBS) at 37 °C, which resemble human physiology. Figure S9 (Supporting Information) shows that the swelling ratios of the dressing gels drastically increased initially on day 1 and then reached a swelling equilibrium of approximately 3000%–4500% around day 2, indicating that all hydrogel dressing samples had stable crosslinked networks that absorbed PBS rapidly. However, with an increase in the silica concentration in the hydrogel dressings, the equilibrium swelling ratios decreased from 4350 ± 360% to 3380 ± 350%, and their swelling ratios reduced from 3900 ± 250% to 2950 ± 240% after the addition of the DNA nanotherapeutic (Figure 5F). A higher silica concentration improved the crosslinking density of the hydrogels, and microstructure observation via FE‐SEM revealed smaller internal pores, which could have increased the number of release pathways and prolonged the release of nanotherapeutics, as shown in Figure 4C.^[^ ^18^ ^]^ This result may be attributed to the strong covalent bonding between silica and FSA, which enhanced the crosslinking density of the bioactive hydrogel networks. Thus, the swelling ratio was inversely proportional to the silica concentration in the hydrogel dressings; similar swelling trends caused by silica concentration have been reported by studies on other polymeric and hydrogel matrices.^[^ ^121^, ^122^, ^123^, ^124^, ^125^ ^]^ Although the addition of both organic and inorganic nanotherapeutics decreased the swelling ratios of the hydrogel dressings, the bioactive hydrogel dressings had sufficient water absorption ability due to SA's superior water retention capacity, which should maintain a moist wound environment and protect wounds from severe infections.

The biodegradability of hydrogels during wound healing is an essential parameter in 3D‐printed tissue engineering because they act as ECMs that provide space for cell growth and mechanical support for regenerated tissues.^[^ ^12^, ^126^ ^]^ Therefore, the degradation profiles of the hydrogel wound dressings were characterized. As shown in Figure S10 (Supporting Information), the degradation rates of all hydrogel dressings in PBS progressively decreased, and all samples exhibited weight losses of under 50% by the end of week 8. These degradation rates are acceptable in wound healing applications. However, contrary to the swelling ratios, the degradation rates in week 8 only slightly increased, even as the silica concentration in the hydrogel dressings increased from 0 wt% to 30 wt%, demonstrating an insignificant resistance to degradation (Figure 5G). The lack of any significant difference between the degradation rates of the samples in week 8 might be attributed to the following reason. Unlike other nanomaterials, the silica NPs in the hydrogel dressings, derived and biomineralized via the sol‐gel process, degraded by themselves, thus accelerating the hydrogel dressings' weight loss (as expected) during the long‐term degradation test. The hydrogel dressings containing DNA nanotherapeutics degraded more rapidly compared with the hydrogel dressings without DNA owing to the release of DNA during the two initial weeks. Thus, the slope of the degradation rate of the hydrogel dressings with silica and DNA was steeper than that of the degradation rate of the hydrogel dressings with silica only. However, after three or 4 weeks, the degradation gap between the hydrogel dressings with and without DNA decreased and overlapped around week 8. The swelling and degradation performance of the hydrogel dressings was observed; they swelled significantly and reached a near‐equilibrium state by the second day. The equilibrium of the swelled state remained for about one to two weeks, after which the swelling gradually degraded.

For effective therapeutic delivery at a local wound site, sustained delivery of molecules is preferred to the excessive burst‐type delivery of nanotherapeutics to maintain an optimal dosage during the wound treatment period.^[^ ^108^ ^]^ To monitor the DNA and silica release behaviors of the dressings, we measured the released DNA using the nanodrop method, and the collective Si ion release was assessed via ICP‐MS. As shown by the cumulative DNA release profile in Figure 5H, all hydrogel dressings exhibited a typical burst‐initiated release profile, with the DNA release being completed within approximately 14 d. In particular, DNA@FSA exhibited a higher initial‐burst release than the other samples; its release rate reached 70% within the first 12 h and further increased to over 80% within 2 d, whereas only 20% of residual DNA was released thereafter. On the contrary, the hydrogel dressings with silica presented sustained release with steady delivery. The DNA‐bSi30@FSA showed a two‐step DNA release profile; a small initial‐burst delivery (less than 50% released) occurred within2 d, followed by a slower, steadier release for the rest of the observation period. A week into the release test, the hydrogel dressing still had about 10% of the loaded DNA, whereas the DNA loaded in FSA had been almost completely released. This significant extension of the DNA nanotherapeutic release depended on the crosslinking network enhanced with the biomineralized silica. The silica covalently bonded by GPTMS with alginate and the biomineralized silica on DNA significantly retarded the DNA release from the bioactive hydrogel dressings. Their small pore size also minimized the loss of DNA. Considering the angiogenic ability and collagen deposition effects of Si^4+^, the ion release of all hydrogel dressings was also investigated through ICP‐MS (Figure 5I). The Si ions from all hydrogel dressings except FSA were released linearly with the immersion time. In the case of FSA, relatively few Si ions were almost released on day 1 owing to the introduction of GPTMS to alginate. In the initial stage (up to day 5), the other hydrogel dressings exhibited sustained Si ion release rates following a high‐to‐low order of silica concentration; the quantity of released Si^4+^ ions was almost proportional to the silica concentrations of the hydrogel dressings. After one week of immersion, the Si^4+^ ion release slowed down. Interestingly, the hydrogel dressings with DNA showed similar release behavior trends but with a slightly reduced release rate of Si^4+^ ions similar to the decreased initial burst of DNA in the hydrogel dressing with 30 wt% silica (Figure 5H). Release profiles from previous studies showing similar trends suggest that hydrogels with protein‐ or peptide‐based therapeutics have a low release rate due to their higher crosslinking density resulting from their double networks or the physical entanglement between the therapeutics and the hydrogel chains, which enables prolonged release kinetics.^[^ ^36^, ^108^ ^]^ Our findings clearly demonstrated that the high crosslinking density of the hydrogel dressings containing high silica concentrations and DNA hindered the rapid loss of DNA and Si^4+^ ions, respectively. The DNA and Si^4+^ ions played roles in suppressing the initial burst and maintaining sustained release independently while the ions performed a synergistic role. This prolonged release of both DNA and Si^4+^ ions from the bioactive hydrogel dressings may improve synergistic bioactive functions, including ROS scavenging and inflammation reduction.

### 2.4 In Vitro Biocompatibility of 3D‐Printed Hydrogel Dressings

The viability of L929 mouse fibroblasts with various concentrations of DNA in culture media was assessed using a cell counting kit 8 (CCK‐8) to evaluate the cytotoxicity and biocompatibility of the DNA nanotherapeutic, as shown in Figure S11A (Supporting Information). No sample group exerted any cytotoxicity on the fibroblasts on day 1, and the relative cell viability of all DNA groups was higher than those of the no‐DNA group. The cell viability of all groups increased greatly from day 1 to day 5. Fibroblasts cultured in media containing DNA concentrations of 25, 50, and 100 µg mL^−1^ had higher cell proliferation rates than the untreated group. A high DNA concentration (500 µg mL^−1^) inhibited the proliferation of the fibroblasts, indicating that an appropriate concentration of DNA needs to be selected to promote fibroblast growth and improve cytocompatibility in closed systems. On day 5, the fibroblasts of all DNA concentration groups proliferated with significant differences, but the degree of significance was lower than that on day 3 due to overpopulation within the wells. To investigate the role played by the DNA nanotherapeutic in skin tissue regeneration, we initially explored its chemotactic effect on the L929 mouse fibroblasts. Based on the cytotoxicity and proliferation results, concentrations of 25 and 50 µg mL^−1^ were selected for the DNA as a chemoattractant. According to the chemotaxis assay results in Figure S11B (Supporting Information), the DNA nanotherapeutic could induce mild in vitro fibroblast chemotaxis at the concentrations of 25 and 50 µg mL^−1^, demonstrating that this DNA nanotherapeutic can accelerate wound healing by stimulating the chemotaxis and increasing the proliferation of fibroblasts. Previous studies show that chemotaxis cell migration during diabetic wound healing is an effect of growth factors. In particular, transforming growth factor (TGF) and platelet‐derived growth factor (PDGF) play a chemotactic role by enabling the accumulation of fibroblasts at wound sites.^[^ ^127^ ^]^ Moreover, platelet‐derived organic and inorganic molecules promote chronic wound healing by enhancing cell chemotaxis.^[^ ^128^, ^129^ ^]^ The DNA from salmon sperm aided in wound healing by acting as an adenosine A_2A_ receptor and producing nucleic acids through the salvage pathway. According to these results, this DNA could encourage cell chemotaxis and potently induce their proliferation and migration.

As wound dressings contact blood, an in vitro hemolysis experiment was conducted following a previously reported protocol to analyze the interaction between the bioactive hydrogel dressings and red blood cells (RBCs).^[^ ^130^ ^]^ Triton X‐100 and PBS were used as the positive and negative controls, respectively, to calculate the hemolysis ratio. As shown in Figure S12 (Supporting Information), the hemolysis ratios of all hydrogels were under 0.5%, which met the ASTM standard (<5%; F756:2008); thus, these developed functionalized hydrogels, which had high negative charge densities, had excellent in vitro hemocompatibility and a lower hemolysis percentage than the critical safe range suggested for biomaterial‐induced hemolysis.^[^ ^98^, ^131^ ^]^ Although the specimens had no significant difference and satisfied the ASTM standard, Si@FSA had the highest hemolysis ratio, probably due to the rapid initial burst of Si^4+^ ions. Optical images also revealed that the supernatants of the RBC suspension treated with hydrogel dressings were colorless and transparent, demonstrating good hemocompatibility.

**Figure 6:**
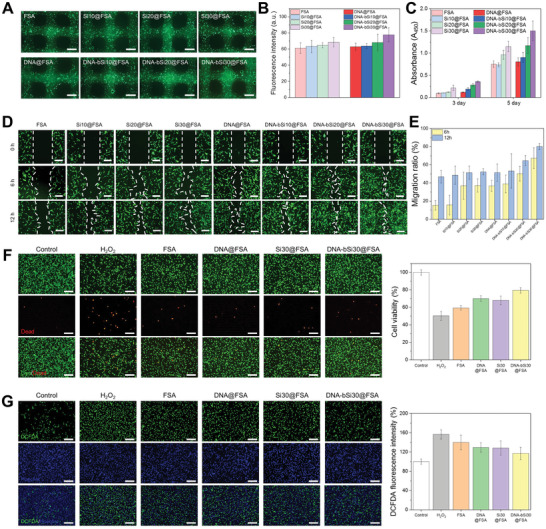
A) Fluorescent images of NIH3T3/GFP‐laden 3D hydrogel scaffolds after 24 h of culturing in medium (scale bar: 1 mm); B) quantification of fluorescence intensity. C) Degree of L929 proliferation in hydrogel dressings after 3 and 5 d of culturing. D) Cell migration images captured after the application of hydrogel dressings onto the scratched areas (scale bar: 200 µm) and E) calculated migration ratios at 6 and 12 h of culturing. F) Evaluation of the antioxidant abilities of the produced dressings after the exposure of L929 to 400 × 10^−6^ m H_2_O_2_, as assessed using live and dead assays (scale bar: 200 µm). G) Verification of the ROS scavenging effect of the hydrogel scaffolds via DCFDA analysis (scale bar: 200 µm).

Bioprinting hydrogels with cells can provide a favorable structural platform for culturing cells originating from soft tissues. As mentioned above, the adjacent subcutaneous tissue has a Young's modulus of 4.5–8 kPa, so we explored the potential of using a complementary network of bioinks to enhance viability of fibroblasts within the 3D‐printed architecture. NIH3T3/GFP fibroblast cells were subjected to direct fluorescence imaging without post‐treatment. After 24 h of culturing, all of the bioprinted hydrogels laden with fibroblasts had smooth surfaces and a square window‐like morphology, suggesting the success of printing cell‐laden multilayer 3D lattices (**Figure** **6**A). The cultured fibroblasts were uniformly distributed throughout the hydrogels. However, the fluorescence intensity of the cell‐laden hydrogels rose with an increase in the silica concentration and the presence of DNA. Thus, all the bioprinted hydrogels could maintain the viability of the NIH3T3/GFP fibroblasts, with the cell‐laden DNA‐bSi30@FSA having the highest cytocompatibility (most of the cells were green). These visual observations were supported by quantitative fluorescence intensity results, which increased with the silica concentration after 1 d of culturing, and the addition of DNA enhanced cell viability (Figure 6B). Extrusion‐based bioprinting generally encounters major challenges in terms of cell viability after printing due to the shear stress applied by the printing nozzle, cytotoxicity of crosslinkers, and incompatibility of hydrogel moduli with soft tissue cells.^[^ ^132^, ^133^ ^]^ By incorporating silica and DNA into hydrogels, we generate cell‐favorable physical and chemical structures with highly tunable moduli and biocompatibility, thereby preserving cell viability after bioprinting.

Biocompatibility is a fundamental requirement of wound dressing materials, and promoting cell regeneration is vital for wound contraction and the subsequent healing. Thus, the cell proliferation activity of the bioactive hydrogel dressings was evaluated using fibroblasts. The proliferation of the L929 mouse fibroblasts in the hydrogel dressings was evaluated using the CCK‐8 assay, and data were collected on days 3 and 5. As shown in Figure 6C, the absorbance value increased remarkably from day 3 to day 5, implying that the mouse fibroblasts rapidly grew on the surfaces of all hydrogel dressings. After 1 d of culturing, no distinct differences in cell proliferation were identified in FSA, Si10@FSA, and Si20@FSA, whereas Si30@FSA had a significantly higher cell proliferation value. The proliferation activity value of FSA with DNA was higher than that of the no‐DNA group; in particular, it gradually increased with the Si content of the hydrogels. This result was attributed to the abundance of silica and DNA nanotherapeutics released by the bioactive hydrogel dressings. The proliferation activity values of the Si20@FSA and Si30@FSA dressings surpassed those of FSA and Si10@FSA on day 5. Notably, the absorbance value of Si30@FSA was approximately 1.5‐fold greater than those of FSA and Si10@FSA. Hence, Si30@FSA had better biocompatibility than FSA owing to the released Si^4+^ ions, and it can aid in the wound healing process without causing long‐term side effects on proliferation, such as nanomaterial cytotoxicity. As for the proliferation values in the with‐DNA group, the DNA‐bSi30@FSA dressing showed a higher proliferation rate compared with the other samples on day 5. This may have resulted from the prolonged release of silica and DNA nanotherapeutics, which regulated the fibroblast cell proliferation, thus improving wound closure. The cell viability and proliferation results showed that the 3D‐printed bioactive hydrogel dressings exhibited satisfactory biocompatibility for use in wound healing.

The cell migration ability of the dressings with DNA was quantitatively evaluated through an in vitro scratch test, where a wound site was recreated within a culturing well to generate a cell‐free zone and observe cell migration to the scratched area. The migration ability of the dressings with DNA was compared with that of the no‐DNA negative control group. The degree of cell migration across the scratched area was compared between the two groups at varying concentrations of silica. As shown in Figure 6D, improved migration with an increase in silica concentration and culturing time was shown compared with the no‐DNA negative control group. The quantified migration ratios (Figure 6E) showed that as the silica concentration increased from 0 wt% to 30 wt% at 6 h, the migration ratio almost doubled. As the culturing time reached 12 h, the migration ratios all exceeded 50%. The experimental group with DNA showed considerably better migration behavior with an increase in culturing time and silica concentration, suggesting that PDRN stimulated and accelerated the fibroblast migration as well. After 6 h of culturing, the migration rate of the 3D‐printed DNA‐incorporated hydrogel dressing without silica was approximately two times higher than that of the no‐silica negative control group. After 12 h of culturing, the migration rate of the DNA‐bSi30@FSA group was the highest among all tested samples and higher by 60% than that of the negative control. These results indicate that on top of the previously confirmed stimulatory effect of silica on fibroblast migration, the incorporation of DNA exerted a synergistic effect by stimulating the cell chemotactic migration activity, which can accelerate wound healing.

Previous studies have shown that the activation of the adenosine A_2A_ receptor prevents ROS generation by regulating the mitochondrial activity.^[^ ^134^, ^135^, ^136^ ^]^ DNA from salmon sperm acts as an adenosine A_2A_ receptor agonist; therefore, it exhibits antioxidant and ROS scavenging effects. Ceravolo et al. stated that DNA protects human corneal endothelial cells against H_2_O_2_‐induced damage; Kim et al. observed DNA antioxidant activity for melanocytes.^[^ ^40^, ^41^ ^]^ The L929 cells were exposed to 400 × 10^−6^ m H_2_O_2_, which is associated with cellular dysfunction and inflammation, to establish an in vitro oxidative stress model and evaluate the antioxidant activity of DNA, which can reduce the damage caused by ROS in wound regions. When the L929 cells were cocultured with H_2_O_2_, approximately half of the cells died during the CCK‐8 assay, suggesting that intracellular ROS successfully reduced L929 cell viability, as shown in Figure 6F. In all hydrogel dressing groups, cell viability recovered to >118% compared with that of the H_2_O_2_‐only‐treated group, and a noticeable increase in cell viability was observed in DNA‐bSi30@FSA compared with FSA, implying that the DNA and biomineralized silica remarkably alleviated oxidative stress, which induced cell death. We further visualized intracellular ROS using DCFDA as an ROS‐sensitive fluorogenic marker. As shown in Figure 6G, a strong green fluorescence of dichlorofluorescein (DCF) was detected in the H_2_O_2_‐treated group, indicating its abundance in the intracellular ROS, whereas a relatively low DCF fluorescence was observed in the control group. Moreover, the DCF fluorescence density of the FSA and Si30@FSA groups was considerably lower than that of the H_2_O_2_‐treated group. The intracellular ROS levels of the L929 cells tested with the DNA‐incorporated hydrogel dressings were significantly lower compared with those of the H_2_O_2_‐treated group and comparable to those of the control group. Thus, the DNA in the hydrogel dressings completely hindered intracellular ROS generation. Overall, our results showed that the DNA‐bSi30@FSA dressing effectively reduced ROS levels and exerted antioxidant activities, consequently improving fibroblast cell survival under oxidative stress damage.

### 2.5 Acute Wound Healing In Vivo

The skin is one of the body's major sensory organs; it has unique immune networks and neuroendocrine functions, plays a crucial role in maintaining body fluids and regulating temperatures, and protects the body from many noxious stimuli, such as pathogens and poisonous substances, and microorganisms as the first line of defense against external stimuli.^[^ ^4^, ^52^, ^137^, ^138^ ^]^ Skin injuries can be caused by burns, accidents, and other diseases and are commonly occurring wounds.^[^ ^1^ ^]^ The following stages are involved in the healing of acute wounds: initial hemostasis, inflammation, proliferation, and remodeling.^[^ ^2^, ^3^, ^4^ ^]^ These stages are overlapping and are crucial for the complete healing of injured skin. The inflammation stage begins immediately after injury; during this stage, many inflammatory cells migrate to the injured site.^[^ ^8^ ^]^ The proliferation stage can be divided into stages as re‐epithelialization, granulation tissue formation, and neovascularization.^[^ ^139^ ^]^ Subsequently, the new tissue comprising collagens and ECMs reorganizes during the final remodeling stage.^[^ ^140^ ^]^ Highly controlled cell‐signaling activities occur during acute wound healing.

Considering the benefits of the fabricated bioactive hydrogels confirmed through in vitro tests, such as hemolysis, chemotactic, and antioxidant properties; ROS scavenging effect; promotion of fibroblast growth and migration; and biocompatibility, the 3D‐printed bioactive hydrogel dressings should shorten wound healing. A full‐thickness acute wound model of mice was tested with saline, FSA, DNA@FSA, Si30@FSA, and DNA‐bSi30@FSA, to compare the healing abilities of the 3D‐printed hydrogel dressings quantitatively under the determined time line (Figure S13A, Supporting Information). The specimens were randomly placed on wounds sites, and optical images of these sites were captured throughout 10 d of healing. Figure S13B (Supporting Information) shows photographs of the wound sites taken at 0, 2, 4, 7, and 10 d. The wounds with hydrogel dressings incorporated with silica or DNA healed better than those in the saline and FSA groups. The saline and FSA groups exhibited slower healing, lacked moisture in the epidermis layer, and showed signs of damage, such as contraction and scab formation.^[^ ^141^ ^]^ On day 10, the DNA‐bSi30@FSA dressing group exhibited almost healed wounds, whereas the saline group without silica and DNA has relatively less‐healed wounds. Therefore, the DNA‐bSi30@FSA dressing had better healing ability, implying a high affinity with cells. The healing rate of the DNA‐bSi30@FSA dressing was higher than those of the other dressings. As shown in Figure S13B (Supporting Information), on day 4 and 10 of healing, the unhealed areas for the DNA‐bSi30@FSA hydrogel dressing group were 48.0% and 22.0%, whereas those for the control group were 88.1% and 57.9%, respectively. Thus, the bioactive hydrogel dressings with DNA and Si^4+^ ions accelerated wound closure and skin regeneration. The hydrogel dressing groups with silica or DNA generally showed rapid healing. However, the individual effectiveness of the released silica and DNA was unclear from the acute wound model. Therefore, further histological study of the 3D‐printed hydrogel dressings was conducted on day 10 to better understand cell involvement in the complex healing process.

Histological sectioning was performed on all 3D‐printed hydrogel dressing groups using hematoxylin and eosin (H&E) and Masson's trichrome (MT) staining to track their compositional differences and understand the interactive healing process in the skin layers (Figure S14, Supporting Information). The wound healing ability of the skin can be monitored using the degree of re‐epithelialization and collagen deposition. Certain physiological activities, such as inflammation, angiogenesis, collagen formation, and contraction, occur as the wound is re‐epithelialized during the early stages of healing. Toward the end of healing, the matrix is remodeled, and granulation tissues are generated.^[^ ^142^ ^]^ In the saline group, the neo‐epidermis around the granulation tissues at the wound site was stretched. Moreover, necrotic and dermal cells were observed in the saline group, and the hair follicles and sebaceous glands at the wound site were sparse. The wounds treated with the other dressing materials were relatively flattened and had more coherent epidermis. In particular, the DNA‐bSi30@FSA dressing site displayed optimal skin healing; its dermis comprised dense connective tissue, a compact epidermis, a well‐ordered dermis, and a thick granulation tissue. The three quantitative indices, including relative dermal thickness, epidermal thickness, and granulation tissue thickness of the DNA‐bSi30@FSA dressing, exhibited the highest values (117.7 ± 7.5%, 94.0 ± 16.3 µm, 654.1 ± 40.2 µm, respectively). Collagen formation in wounded tissue is also an essential signal for the regeneration of damaged tissue. Figure S14 (Supporting Information) also shows the MT staining images; the visualized collagen area (blue) in the saline group was clearly smaller than those for the other groups. Moreover, well‐organized collagens were abundant in the bioactive hydrogel dressing group, which agreed with the H&E staining results suggesting that the DNA‐bSi30@FSA dressing had the thickest granulation tissue. The collagen density of the hydrogel dressing groups increased parabolically from 188.3 ± 11.4% to 282.0 ± 20.1% compared with that of the saline group. Together, these data indicated that the DNA‐bSi30@FSA dressing group exhibited better acute wound healing than the other hydrogel dressing groups.

TGF, MPO, CD31, and VEGF were immunohistochemically stained in the sampled wound sections of the saline and experimental groups to examine the mechanisms of the important biomarkers' and molecules activities that occur in the healing of the acute wounds treated with the hydrogel dressings. TGF‐*β*, which plays a key role in diverse cellular activities, is also an important regulator in many phases of the wound healing process; e.g., it influences cellular activities in the skin, including proliferation, migration, differentiation, and apoptosis.^[^ ^143^ ^]^ It also regulates the remodeling of ECMs and suppresses immune responses.^[^ ^144^ ^]^ The regulatory activity of TGF‐*β* influences the scarring of skin tissues, which may lead to abnormal processes, such as hypertrophic scarring. Among the TGF‐*β* isoforms, TGF‐*β*1 and TGF‐*β*2 exhibit suppressed expression during normal healing, but their expression and signaling are upregulated in hypertrophic scars. In particular, TGF‐*β*1 induces the differentiation of myofibroblasts, which are key potential targets for the treatment of hypertrophic scars and keloids.^[^ ^145^, ^146^ ^]^ With the regulation of TGF‐*β* playing such a critical role in wound healing, many researchers have studied the TGF‐*β* pathway to find potential breakthrough treatments for wound repair. Moreover, TGF‐*β*1 acts as a chemoattractant of various cells, such as endothelial cells, fibroblasts, neutrophils, and monocytes, and induce the release of inflammatory cytokines, including IL‐1b and IL‐6, along with the differentiation of macrophages.^[^ ^147^, ^148^, ^149^, ^150^ ^]^ Therefore, immunohistochemical staining of TGF‐*β*1 was performed (Figure S15, Supporting Information). The saline, FSA, and DNA@FSA groups showed intense TGF‐*β*1‐positive staining, whereas the Si30@FSA and DNA‐bSi30@FSA groups showed a weaker TGF‐*β*1‐positive expression. The TGF‐*β*1‐positive expression of the DNA‐bSi30@FSA group was 10.4%, which was the lowest among all groups. Therefore, the DNA‐bSi30@FSA group significantly relieved inflammation and efficiently inhibited scar formation.

When the skin is injured, inflammatory activities consistently occur during wound healing to resist infection. However, inflammation at wound sites can lead to the fibrotic scarring of the repaired tissue.^[^ ^151^ ^]^ The migration of inflammatory cells, such as neutrophils, to wounded regions can combat pathogen invasion, but it can also delay the healing of the wounded skin and corneal epithelium.^[^ ^152^ ^]^ MPO, a leukocyte‐derived enzyme, can be used to quantify the degree of inflammatory response of skin wounds as it is a positive biomarker for neutrophil expression.^[^ ^153^ ^]^ As shown in Figure S15 (Supporting Information), immunohistochemically stained MPO (green) was observed in the saline group on day 10, demonstrating excellent neutrophils recruitment at the inflammation site. The MPO levels in the FSA group were lower than those in the saline group. However, compared with the MPO values of the Si‐incorporated hydrogels, the relatively high values of FSA indicated a low degree of inflammation reduction. The hydrogel dressings with DNA or silica showed remarkably decreased MPO values because of their ROS scavenging effect and antioxidant ability, which can protect skin tissues at wound sites from oxidative stress.^[^ ^154^ ^]^ In this study, the DNA‐bSi30@FSA group yielded the lowest levels of MPO among all tested groups, indicating the potential synergistic effect of PDRN and biomineralized silica.

Angiogenesis, the formation of new blood vessels, is an important process during wound healing. The roles of blood vessels include transporting essential oxygen, nutrient, and waste products as the tissue regenerates during wound healing. Cluster of differentiation 31 (CD31), a transmembrane glycoprotein with major roles in inflammation, vascular endothelial cell (VEC) migration, and angiogenesis, is a commonly used vascular marker for endothelial cells and cytoprotection, which are crucial for wound healing. CD31 is expressed in the early stages of vascular remodeling. Another important vascular factor is VEGF, a main growth factor for VECs and a potent angiogenic factor; it plays a pivotal role in hematopoiesis, bone regeneration, and wound healing.^[^ ^155^ ^]^ It is present in the vessels during the late stages of healing. In this study, both vascular markers were used to analyze the angiogenic abilities of the bioactive hydrogel dressings. The angiogenesis phenomenon was visually observed in all hydrogel dressing‐treated groups using CD31 and VEGF as markers (Figure S15, Supporting Information). The angiogenesis promotion abilities of both the released DNA and silica from the hydrogel dressings induced blood vessel formation and vascularization. On day 10, the DNA‐bSi30@FSA group exhibited the highest CD31 and VEGF expression among all compared groups, showing a greater tendency of vascularization. From these results, we infer that although DNA and silica individually have angiogenesis abilities, the combination of DNA and biomineralized silica synergistically promoted angiogenesis, thereby further accelerating acute wound healing and regeneration.

### 2.6 In Vivo Chronic Wound Healing in a Diabetic Model

**Figure 7:**
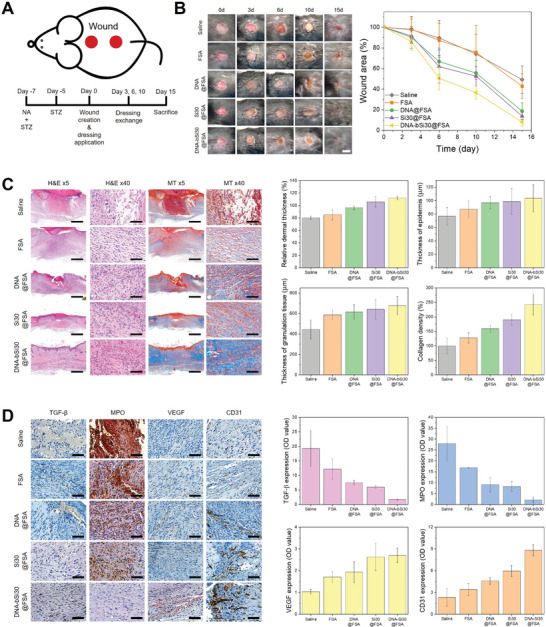
A) Schematic diagram and timeline of the in vivo diabetic‐wound healing experiments. B) Captured images of the wound area after the determined breeding time, and wound‐closing rate calculated from the obtained images (scale bar: 5 mm). C) Histological images of H&E‐ and MT‐stained tissues, and computed relative dermal thickness, epidermal thickness, granulation tissue thickness, and collagen density (scale bar: 500 µm for 5× magnification and 50 µm for 40× magnification). D) Immunohistochemical analysis of the extracted tissues for visualizing the degree of inflammation (TGF‐*β* and MPO) and angiogenesis (VEGF and CD31) (scale bar: 50 µm).

Motivated by the results obtained from the acute wound model, we investigated the chronic wound healing abilities of the 3D‐printed bioactive dressings in a diabetic mice model (**Figure** **7**A). Optical images of the wounds, closure traces, and wound areas during wound healing are shown in Figure 7B, and their progress was monitored by examining the wound areas for 15 d. On day 3, the 3D‐printed hydrogel dressing groups containing DNA or silica revealed accelerated wound closure. On day 6, the remaining wound of the DNA‐bSi30@FSA dressing group was remarkably reduced, whereas the wound areas of the control, FSA, DNA@FSA, and Si30@FSA dressing groups were still as large as 89.7%, 87.3%, 66.8%, and 61.9%, respectively. On day 6, a large part of the chronic wound in the DNA‐bSi30@FSA dressing group was healed, with a remaining wound area of 50.5%. Larger wound areas remained in the saline and FSA dressing groups, whereas Si@FSA and DNA@FSA showed quicker healing rates than the two other two dressing groups, with a wound closure ratio of approximately 50%. This trend was observed from day 3 to day 10. Finally, on day 15, the wounds treated with the DNA‐bSi30@FSA, Si30@FSA, and DNA@FSA dressing groups showed faster healing than those of the saline and FSA dressing groups. However, the wound closure ratio of the DNA‐bSi30@FSA dressing group was only 8.0%, whereas the wound closure rates of the Si30@FSA and DNA@FSA dressing groups were still 18.7% and 13.9%, respectively. Thus, the 3D‐printed DNA‐bSi30@FSA dressing could significantly enhance wound healing in a chronic wound in diabetic mice by enhancing the synergistic bioactive functions of DNA and biomineralized silica nanotherapeutics.

Histological evaluation of wound tissues was performed on day 15 to examine diabetic wound healing closely. The saline group barely showed epithelial regeneration. The granulation tissues were still loose (not filled) owing to incomplete dermis formation as the inflammation stage progressed. The hydrogel dressing‐treated groups showed elemental epithelial structures, appropriate collagen fibers, and fibroblasts in the diabetic wounds (Figure 7C). The wound of the DNA‐bSi30@FSA dressing group had the thickest dermal layer (112.3 ± 3.3%), which was significantly different from those of the saline and FSA groups. The dermis, comprising the papillary and reticular dermis (beneath the epidermis), plays a primary role in cutaneous nerve signal sensing and thermoregulation because it contains nerve endings, hair follicles, sweat and sebaceous glands, and blood vessels.^[^ ^4^ ^]^ Therefore, according to our results, skin function can be recovered by the regenerated dense dermis by the DNA from salmon sperm and the biomineralized silica. As the regenerated granulation tissues rapidly became mature dermis tissues through the bioactive hydrogel dressing treatment, the main component of the dermis, such as hair follicles and collagens, increased in abundance in the DNA‐bSi30@FSA dressing group, as shown by the H&E and MT staining results. We hypothesized that the groups would significantly differ in epidermis thickness, as their wound healing abilities noticeably varied. However, contrary to our hypothesis, no significant difference was identified in the epidermis thickness of the wounded skin between the different groups. The epidermis thickness generally increases with epidermal hyperplasia as epithelial cells migrate to the wounded area. Then, toward the later stages of wound healing, the epithelium thickness returns to normal. Although the DNA@FSA, Si30@FSA, and DNA‐bSi30@FSA samples showed significantly enhanced wound healing abilities in wound closure tests, the lack of a difference in epidermis thickness may have been because these wounds were undergoing different stages of healing when they were examined; those experiencing accelerated wound repair were in a later stage of healing compared with the wounds of the saline and FSA groups, which were healing at a slower rate and were thus in relatively earlier stages of wound healing during the examination.^[^ ^156^ ^]^

Additional TGF‐*β*, MPO, VEGF, and CD31 staining was performed using the same method as that for the acute wound model to evaluate the inflammatory and angiogenesis responses of the diabetic wound regions under the different dressing treatments. Unlike the healing of acute wounds, the healing of a diabetic wound is generally stalled in the inflammation stage owing to excessive ROS production and pro‐inflammatory cytokines, which are secreted by inflammatory cells (neutrophils and M1 phenotype macrophages).^[^ ^157^ ^]^ Uncontrolled ROS exerts oxidative stress on various biomolecules, such as cellular DNA, proteins, lipids, and the skin cells within chronic wounds that contribute to inflammation‐related pathologies, consequently leading to the long‐term incurability of chronic wounds. In particular, neutrophils are impaired by chronic inflammation in the environment of long‐term hyperglycemia, thus inducing ROS secretion.^[^ ^158^ ^]^ Therefore, the antioxidant capacity of the endogenous system is insufficient for reducing such oxidative stress damage, and exogenous antioxidants should be used to scavenge the accumulated ROS. TGF‐*β* and MPO are reliable biomarkers of the activation of inflammatory cells, such as neutrophils. The trend observed via TGF‐*β* and MPO immunochemistry staining in chronic wound healing in diabetes was comparable with the results obtained from the acute wound model. The TGF‐*β* and MPO levels in the 3D‐printed FSA dressing with DNA and/or silica groups were lower than those of the non‐hydrogel‐treated group, as shown in Figure 7D. However, the FSA dressing with DNA, which exhibited ROS scavenging, had lower TGF‐*β* and MPO expression levels in the diabetic wound model than in the acute wound model. Hence, the DNA effectively suppressed inflammation by relieving oxidative stress, and the ROS scavenging and biomineralized silica helped the DNA alleviate the inflammatory response.

Vascular dysfunction is a complication of non‐healing chronic wounds in diabetes. Hyperglycemia leads to the contraction of blood vessels and impedes neovascularization, which can hamper wound healing by inhibiting endothelial cell function and oxygen supply.^[^ ^159^ ^]^ Moreover, deficient angiogenesis and excessive oxidative stress, the pathological factors of impaired diabetic wound healing, are correlated with each other because ROS accumulation inhibits angiogenic responses and results in endothelial dysfunction.^[^ ^160^, ^161^ ^]^ Diabetes causes patients to be more prone to oxidative stress and diseases originating from inflammation, as the ample supply of glucose leads to enhanced endothelial cell nitric oxide synthase activity and superoxide production, which promote the active formation of hydroxyl radicals, H_2_O_2_, and peroxynitrite. Furthermore, angiogenesis acceleration and ROS scavenging promote wound repair and enhance the quality of healing of various types of skin wounds, including diabetic wounds.^[^ ^162^, ^163^, ^164^, ^165^, ^166^, ^167^ ^]^ Therefore, in our hydrogel design, we used DNA and silica, which have good angiogenesis effects. We found out that bioactive hydrogel dressings incorporated with DNA or silica independently induced neovascularization at wound sites in diabetic mice, as shown in Figure 7D. In particular, the DNA‐bSi30@FSA group had the highest CD31 and VEGF expression values among the experimental groups, showing greater vascularization tendency. Thus, the biomineralized silica and DNA synergistically improved new blood vessel growth, that is, good angiogenic ability. The above results showed that the 3D‐printed DNA‐bSi30@FSA dressings have various advantages. The porosity of their 3D structures facilitates the absorption of exudates and blood from the wounded region and provides an environment conducive to fibroblast growth on the microscale. In addition, DNA from salmon sperm and DNA‐induced biosilica enhance the mechanical properties of hydrogels such that they exhibit good shape fidelity and printability during 3D printing, and protect skin surface wounds from the internal and external mechanical deformations that can occur during wound repair. Lastly, the 3D‐printed bioactive hydrogels can sustain the release of both the DNA and biomineralized silica, thus generating beneficial therapeutic effects, such as hemostatic, chemotactic, and antioxidant properties; ROS scavenging effect; fibroblast growth promotion and migration; anti‐inflammation effects; and angiogenesis. These hydrogels ultimately accelerate acute and diabetic wound healing.

## 3 Conclusions

In summary, our results reveal a new paradigm in 3D‐printed bioactive hydrogel dressings made from marine‐derived and ‐inspired functional materials for diabetic wound healing. With this strategy, DNA from salmon sperm and DNA‐induced biosilica were incorporated into 3D‐printed alginate hydrogel dressings in a fast yet intuitive manner. These dressings provided appropriate porosity, facilitating the absorption of exudates and blood at wound sites, and mechanical tunability, for good shape fidelity and printability during AI‐based 3D printing, thus protecting the wounds from the internal and external mechanical deformations that can occur during wound healing. Moreover, both the DNA and biomineralized silica acted as nanotherapeutics, enhancing the biological activities of the 3D‐printed bioactive hydrogel dressings regarding ROS scavenging, angiogenesis, and anti‐inflammation activity, thus ultimately accelerating acute and diabetic wound healing. These bioinspired 3D‐printed hydrogels with a DNA‐induced biomineralization strategy are a promising functional platform for clinical application in acute and chronic wound repair.

## 4 Experimental Section

### Materials

The following items were purchased from Sigma Aldrich, USA: Salt formed sodium alginate (SA), calcium chloride (CaCl_2_), (3‐glycidyloxypropyl)trimethoxysilane (GPTMS), tetramethyl orthosilicate (TMOS), 2′,7′‐dichlorodihydrofluorescein diacetate (DCFH‐DA), Hoechst 33342, nicotinamide (NT), streptozotocin (STZ), eosin Y solution (HT110332), citrate buffer solution, 0.09 m, antibiotic antimycotic solution (100×), hydrogen peroxide solution (H_2_O_2_, 30% (w/w) in H_2_O). Gibco, USA: fetal bovine serum (FBS), 0.05% Trypsin‐EDTA (1X). Genoss, South Korea: polydeoxyribonucleotide (PDRN, DNA). Dae‐Jung, South Korea: hydrochloric acid (HCl). Fisher Scientific, USA: Dulbecco's phosphate buffered saline (DPBS, pH 7.4). Welgene, South Korea: Alpha MEM. Dongin‐LS, South Korea: D‐Plus CCK cell viability assay kit. TOYO, Japan: Advantec Syringe Filter CA 25 mm. Invitrogen, Thermofisher Scientific, USA: calcein acetoxymethyl ester (Calcein AM), Hoechst 33342. Abcam, UK: DCFDA/H2DCFDA – Cellular ROS Assay Kit (ab113851), Mayer's, modified hematoxylin (ab220365), Masson's Trichrome Stain Kit (M‐T, ab150686), anti‐VEGF Receptor 1 (ab32152) antibody, Anti‐TGF *β*1 (ab215715) antibody, Anti‐Myeloperoxidase (MPO, ab208670) antibody, anti‐*α*‐smooth muscle Actin (SMA, ab5694) antibody.

### Synthesis of Nature‐Derived and ‐Inspired Bioactive Hydrogel Inks

Functionalized alginate hydrogel (FSA) was formed by reacting alginate and GPTMS according to literature.^[^ ^62^ ^]^ Briefly, a calculated amount of GPTMS in 3 w/v% alginate in distilled water (DW) was added to 0.01 n HCl (pH = 5) and stirred at 50 °C for 72 h. The final amount of GPTMS was set to achieve a molar ratio of alginate to GPTMS as 10:1. Then, a predetermined amount of DNA was introduced and stirred for an additional 1 h. DW and 1 n HCl were combined to hydrolyze TMOS for 1 h for biomineralization process. The hydrolyzed TMOS was added to the DNA@FSA solution and mixed for another 1 h to homogenize, co‐condense, and biomineralize. The biomineralized silica contents of the fabricated bioactive FSAs with DNA were 0, 10, 20, and 30 w/w%, and the samples were named DNA@FSA, DNA‐bSi10@FSA, DNA‐bSi20@FSA, and DNA‐bSi30@FSA, respectively, whereas the samples were named FSA, Si10@FSA, Si20@FSA, and Si30@FSA when the silica contents of the fabricated hydrogels without DNA were 0, 10, 20, and 30 w/w%, respectively. DNA was loaded in each sample at a concentration of 50 µg mL^−1^.

### Measurement of the Zeta Potential and the Size Distribution

Zeta potential and size distributions of DNA and silica were measured by dynamic light scattering (DLS; Zetasizer Nano ZS, Malvern Instruments Ltd., Malvern, UK). Analysis was carried out using 633 nm laser with a scattering angle of 173° at 25 °C. Five measurements were averaged to get the data. The procedure to detect synthesized DNA‐bSi@FSA ink was same to the procedure above.

### Cryo‐TEM Analysis to Characterize Biomineralization

The morphology of DNA was examined using cryogenic transmission electron microscope (cryo‐TEM). The holey carbon grid (Quantifoil R1.2/1.3, 200 mesh Cu, Structure Probe Inc., USA) which was treated with glow discharger at 15 mA for 60 s to increase the loading efficiency was loaded inside the Vitrobot Mark IV (Thermofisher, USA, SNU CMCI) at 15 °C with 100% humidity. 3 µL of the diluted sample solution was mounted and excessive amount of the solution was blotted for 4 s in force 1 and specimens were plunge frozen immediately in liquid ethane to prepare cryo‐TEM sample. Cryo‐TEM sample was loaded to a cryo‐EM holder (626 single tilt cryo‐EM holder, Gatan, USA) with temperature maintained at nearly ‐180 °C. The cryo‐EM holder was loaded into a TEM (JEM‐2100F, JEOL, Japan) and images of the frozen hydrogel were acquired at an acceleration voltage of 200 kV.

The internal structure of the DNA‐bSi30@FSA was analyzed using transmission electron microscopy (TEM, JEM‐2100F, JEOL, Japan) coupled with energy‐dispersive X‐ray spectroscopy (EDS) at an acceleration voltage of 200 kV. TEM sample was prepared by homogenizing the hydrogels to granules and diluted with DW water before loading on a holey carbon grid (Quantifoil R1.2/1.3, 200 mesh Cu, Structure Probe Inc., USA). TEM sample was dried under vacuum for 24 h before the observation. The morphology comparison of the silica in Si30@FSA and DNA‐bSi30@FSA was also examined using cryo‐TEM.

### NMR Analysis to Characterize Chemical Bonding of Hydrogel Inks

The chemical bonding of synthesized hydrogel inks and the rate of silica condensation in hydrogel inks were investigated using 600 MHz ^1^H‐nuclear magnetic resonance spectrometry (NMR, AVANCE 600, Bruker, Germany) equipped with 5 mm BBO BB‐H&F‐D CryoProbe Prodigy at liquid state using D_2_O as solvent. Additional solid state NMR analyses were performed by 500 MHz ^13^C‐nuclear magnetic resonance spectrometry (NMR, Avance III HD, Bruker, Germany) equipped with 2.5 mm CP MAS Probe.

### XPS Analysis to Characterize Cross‐Linked Network of Hydrogel Inks

The chemical composition and the presence of cross‐linked network in 3D‐printed hydrogel dressings were analyzed using X‐ray photoelectron spectrometry (XPS, Thermo ESCALAB 250XI, Thermo Fisher, USA) with monochromatic Al K*α* X‐ray source. Analyzed spot size was 500 µm for all tests.

### Rheological Characterizations of Hydrogel Inks

The rheological behaviors of the Si@FSA and DNA‐bSi@FSA inks with different silica and DNA contents were examined using a rheometer (Discovery HR 30, TA Instruments, USA). 1 mL of the hydrogel inks was positioned between 40 mm diameter parallel plates, and frequency sweep tests were carried out in the range of 0.1–100 rad s^−1^ under a constant strain of 1%. As a function of frequency, the storage modulus (*G*′) and loss modulus (*G*″) were measured. The shear viscosity of each hydrogel inks was also measured at shear rates from 0.1 to 100 s^−1^.

### Machine Learning‐Based 3D Printing

A machine learning model was developed by using MATLAB software (The MathWorks, Inc. USA) with Statistics and Machine Learning Toolbox which provides various algorithm to make models in this work. The data with 1, 2, 3, and 4 w/v% of FSA concentration in DNA@FSA were used to train and validate the machine learning model and the data with 2.5 w/v% of FSA concentration in DNA@FSA were used to evaluate the trained model. FSA concentration, nozzle size, temperature and pneumatic pressure were considered as input variables and the score values as output variable. The values of nozzle size, temperature and pneumatic pressure were applied after logarithmic transformation to match the range with FSA concentration. Fivefold cross validation scheme and the optimizable Gaussian process regression (GPR) model were applied to predict the score of the experimental 3D printing data. In order to optimize hyperparameters of the model Bayesian optimization was used, and finally the constant base function, nonisotropic rational quadratic kernel function, kernel scale of 0.17363 and sigma value of 2.2405 were adopted for the trained GPR model.

### 3D Printing of Hydrogel Dressings

A mesh type wound dressing model was designed using SolidWorks 2017 software (Dassault Systemes SolidWorks Corp., USA) and exported as a STL file and sliced by Organ regenerator (ROKIT healthcare, Korea) g‐code comments to interface with the 3D printer hardware (ROKIT healthcare, Korea). Prepared alginate‐based hydrogel inks were mixed with 0.3 w/v% CaCl_2_ solutions to partially cross‐link hydrogel inks for printability. Pre‐cross‐linked hydrogel inks were printed using a bio‐printer (Dr. INVIVO 4D6, ROKIT healthcare, Korea) with a 22G (0.4 mm) nozzle at 37 °C and determined pneumatic pressure to create a 10 × 10 × 2 (mm^3^) sample. Then, 3D‐printed hydrogel inks were post‐cross‐linked by 1 w/v% CaCl_2_ to improve the interface adhesion between layers. Complete gelation of the 3D‐printed hydrogel dressings was acquired after 10 min at room temperature. 3D‐printed DNA‐bSi30@FSA dressings were characterized using optical images to confirm printability.

### FE‐SEM to Characterize Microstructure of Hydrogel Dressing

DNA‐bSi30@FSA dressings were characterized using field emission scanning electron microscopy (FE‐SEM; S‐4800, HITACHI, Japan) and EDS spectrometer to identify changes in the structure and elemental distribution, respectively. Air‐dried hydrogel scaffolds and freeze‐dried hydrogel dressings with different concentrations of silica were prepared, then their surface and cross‐sectional morphologies were observed. Using ImageJ software (National Institute of Health, Maryland, USA), the average pore size of hydrogel dressings was calculated.

### TGA Analysis

Thermal analysis of 3D‐printed hydrogel dressings was examined using a thermogravimetric analyzer (TGA2, Mettler‐Toledo, Germany) with a heating rate of 10 °C min from 30 to 900 °C. A flow rate of 50 mL min^−1^ in an environment of N_2_ was used to determine the thermal prepared 5 mg of sample.

### XRD Analysis

Using Cu‐K radiation at room temperature, the phase and crystallinity were investigated using an X‐ray diffractometer (XRD, MimiFlex600, Rigaku Inc., Tokyo, Japan). All the hydrogel samples were continuously scanned from 10 to 70° in 2*θ* at 1° min^−1^.

### FT‐IR Spectrophotometry

The chemical bonds in 3D‐printed hydrogel dressings were confirmed using Fourier‐transform infrared (FT‐IR) spectroscopy (Cary 630 FTIR, Agilent, USA). Between 650 and 4000 cm^−1^, FT‐IR spectra of alginate, FSA, DNA‐bSi10@FSA, DNA‐bSi20@FSA, DNA‐bSi30@FSA, and silica were obtained.

### Rheological and Compressive Mechanical Characterizations

The rheological mechanical properties of 3D‐printed the Si@FSA and DNA‐bSi@FSA dressings with different silica and DNA contents were examined using a rheometer. The oscillation frequency tests were carried out under a continuous load of 1% in the frequency range of 0.1–100 rad s^−1^ after each 3D‐printed hydrogel dressing was positioned between 20 mm diameter parallel plates. A function of frequency was used to measure the storage modulus (*G*′) and loss modulus (*G*′′). For comparison, the storage modulus and loss modulus of the hydrogel dressings with and without DNA were also obtained at 10 Hz. Using a universal testing machine (MTDI INC., Korea), compressive properties of hydrogels were assessed. 3D‐printed hydrogels with an 18 mm diameter and 6 mm height were preconditioned by keeping the samples in the test temperature for 1 h. During testing, a 1000 N load cell was used to maintain a crosshead speed of 2 mm min^−1^.

### Swelling Test

Swelling ratio in terms of immersion time was assessed by submerging in DPBS at 37 °C. The weights of the hydrogels at the equilibrium state were measured after defined time intervals. Following the equation, the swelling ratio was also computed:

(1) $$\mathrm{Swelling}\;\mathrm{ratio}\;\;\left(\%\right)=\;\frac{W_{\mathrm{wet}}-W_{\mathrm{dry}}}{W_{\mathrm{dry}}}\times 100$$

### Degradation Profile

The cross‐linking effect of silica and PDRN was investigated on the degradation profiles of 3D‐printed the Si@FSA and DNA‐bSi@FSA dressings with different silica and DNA contents. The DPBS‐immersed hydrogels were weighed at predetermined intervals. In addition, the degradation ratio was calculated following the equation:

(2) $$\mathrm{Degradation}\;\mathrm{ratio}\;\;\left(\%\right)=\;\frac{W_{\mathrm{dry}}}{W_{\mathrm{wet}}}\times 100$$

### In Vitro DNA Release Behavior

DNA release behavior from the hydrogel dressings was measured by using UV‐Vis spectroscopy (Microplate Multi Reader‐Synergy H1, BioTek, USA) at 258 nm. A standard curve was created by measuring a specific quantity of DNA dissolved in DPBS. An absorbance peak at 258 nm was seen in a typical curve of the DNA absorbance released from the hydrogel. The hydrogel dressings were in contact with a total of 2 mL of DPBS solution. A 0.22 µm syringe filter was used to filter the solutions to remove any impurities. Then, the absorbance was used to quantify the filtrates.

### In Vitro Si Ion Release Behavior

Inductively coupled plasma mass spectrometry (ICP‐MS; ICAP Q, Thermo scientific, USA) was used to measure the concentration of the released Si ions from each sample at 37 °C using the 3D‐printed the Si@FSA and DNA‐bSi@FSA dressings with different silica and DNA contents with 26 mm diameter and 15 mm height. The extracted solution was collected at determined time after immersion and replaced with fresh DPBS.

### Cell Cytotoxicity and Proliferation Assay

The viability of L929 in culture medium with various concentration of DNA from 0 to 500 µg mL^−1^ on 1, 3, and 5 d was detected by CCK‐8 Kit. The fibroblasts of each PDRN concentration group were cultured in medium containing 10% CCK‐8 at 37 °C for 3 h, and a microplate reader (Infinite F50, Tecan, Switzerland) was used to measure the absorbance of the medium at 450 nm.

### Chemotaxis Assay

In vitro 2D chemotaxis assays were performed by the µ‐slide chemotaxis chamber system (Ibidi GmbH, Martinsried, Germany) according to the protocol provided by the manufacturer. Briefly, the cell culture medium solution suspended with fibroblasts was loaded into the chemotaxis channel, and the chamber systems were incubated for 4 h at 37 °C in 5% CO_2_ for collagen gelation. After incubation, one side of the chemotaxis chamber was filled with cell culture medium with various concentration of DNA from 0 to 50 µg mL^−1^ as a chemoattractant, whereas the other side of the chemotaxis chamber reservoir was filled with cell culture medium alone. The chemotactic index was calculated by dividing the number of fibroblasts in the presence of chemoattractant (DNA) by the number of migrated cells in the absence of the chemoattractant (control).

### In Vitro Hemolysis Test

The red blood cells (RBCs) from centrifuged whole blood of healthy volunteers (KOREAN Red Cross Blood Services, Seoul, Korea) were used to evaluate the hydrogel dressing‐mediated hemolysis according to the standard protocol ISO10993.4.^[^ ^129^ ^]^ Ethical clearance was obtained from Institutional Review Board (IRB) of The Catholic University of Korea (IRB No. 1040395‐202203‐HBM‐903). 5 mL of 0.9 w/v% NaCl solution and 0.2 mL of whole blood were combined to create a diluted whole blood solution. FSA, Si30@FSA, DNA@FSA, and DNA‐bSi30@FSA dressings were placed in abovementioned solution, and kept for 30 min at 37 °C. After that, the hydrogel dressings were taken off, centrifuged for 5 min at 3000 rpm, and the supernatant was collected and analyzed by measuring absorbance at 545 nm with a microplate reader (Synergy H1 Hybrid Multi‐Mode Reader, BioTek, USA). According to the following formula, hemolysis was determined.

(3) $$\mathrm{Hemolysis}\;\;\left(\%\right)=\;\frac{{\mathrm{OD}}_{\mathrm{sam}}-{\mathrm{OD}}_{\mathrm{neg}}}{{\mathrm{OD}}_{\mathrm{pos}}-{\mathrm{OD}}_{\mathrm{neg}}}\times 100$$

where the absorbance of solutions containing experimental samples, negative control (Triton X‐100), and positive control (DPBS) are designated as OD_sam_, OD_neg_, and OD_pos_, respectively.

### Bioprinting

For the cell‐laden bioprinting, bioink was prepared by mixing 5 × 10^6^ cells mL^−1^ of NIH3T3/GFP cell (CRL‐1730ATCC, USA) suspension with the pre‐cross‐linked hydrogel inks for each condition. Bioprinting with cell‐laden bioink in 10 × 10 × 2 (mm^3^) was conducted following identical procedure as described above. Cell‐laden hydrogel dressings were cultivated for 24 h at 37 °C in a humidified incubator with an air environment containing 5% CO_2_, followed by fluorescence imaging. Using ImageJ software, the green fluorescence intensity of bioprinted hydrogels was calculated.

### Cell Migration

An in vitro cell migration assay was carried out in accordance with prior literature descriptions.^[^ ^103^ ^]^ On a culture plate, 5 × 10^4^ cells mL^−1^ of NIH3T3/GFP cells were distributed and cultivated for 24 h. A mechanical scratch was applied at the center of the wells. The gap width that resulted was the same as the tip end's outer diameter. The well was scratched, and the detached cells were removed by washing with fresh medium. Then, the various 3D‐printed hydrogel dressings were placed on the scratched cellular membrane. The well without the hydrogel dressings was set as the control group. Using an optical microscope (ECLIPSE Ti2, Nikon, Japan), optical fluorescence images of each well were acquired after 6, 12, or 24 h of culture. Image J software was used to calculate the ratios of the original scratch (*A*_0_) and healing scratch (*A*_t_). The migration ratio (*A*) was determined using the following equation:^[^ ^103^ ^]^

(4) $$\mathrm{Migration}\;\mathrm{ratio},\;A\;\;\left(\%\right)=\;\frac{A_{o}-A_{t}}{A_{o}}\times 100$$

### Cell Proliferation Assay

Viability of the cocultured L929 cells in the various 3D‐printed hydrogel dressings on 3 and 5 d was detected by CCK‐8 Kit. The hydrogel dressings of each group with *n* = 4 were cultured in a medium containing 10% CCK‐8 at 37 °C for 3 h, then the absorbance of the medium at 450 nm was measured using a microplate reader (Infinite F50, Tecan, Switzerland).

### ROS Scavenging Assays

ROS protective effect of various 3D‐printed hydrogel dressings to fibroblasts was observed by live/dead staining assay to evaluate the antioxidant properties. In order to obtain the extract medium, the 3D‐printed hydrogel dressings (FSA, Si30@FSA, DNA@FSA, and DNA‐bSi30@FSA dressings) were treated in a medium with an air environment containing 5% CO_2_ for 24 h. L929 cells with a cell density of 2 × 10^4^ cells mL^−1^ were seeded in tissue culture plate and incubated under 5% CO_2_ at 37 °C for 24 h. Following the incubation, the culturing media was exchanged to the previously extracted media containing 400 × 10^−6^ m H_2_O_2_ were introduced. Following 10 h of incubation, the culture media was discarded, and the L929 cells were washed carefully with fresh medium. Next, the live/dead cell staining assay was used to evaluate the cell viability treated with different hydrogel dressings.

The effect of 3D‐printed hydrogel dressings on ROS level in L929 cells with oxidative stress was evaluated using the DCFH‐DA assay. Using the same method as same the abovementioned antioxidant assay test, the extract medium was obtained and the L929 cells were cultured. The culturing medium was replaced with the extract media containing 400 × 10^−6^ m H_2_O_2_ and the L929 cells were incubated for 1 h. The L929 cells were stained with the mixture of DCFH‐DA and Hoechst 33342 at 37 °C for 1 h in dark. The ROS scavenging effect within the L929 cell was evaluated by the fluorescence intensity of 2′‐7′‐dichlorodihydrofluorescein, a high fluorogenic probe obtained by the oxidation of DCFH‐DA resulting from the generated ROS. The nuclei stained with Hoechst 33342 fluorescent DNA‐binding dye were evaluated using a microplate reader (Synergy H1 Hybrid Multi‐Mode Reader, BioTek, USA, Ex. 495 nm; Em. 529 nm) and fluorescence microscope (Nikon ECLIPSE Ti2, Nikon, Japan).

### In Vivo Assessment

Ethical clearance was obtained from the Institutional Review Board (IRB) of The Catholic University of Korea (IRB No. 1040395‐202107‐HBM‐901). Male BALB/c mice (8 weeks, body weight 20 g) and Male C57BL/6 mice (8 weeks, body weight 20 g) were purchased from Orientbio. The mice were kept in an animal facility with a 20–23 °C temperature range, a humidity range of 40–60%, and a 12 h light/dark cycle. Fasting was kept up for 16 h prior to introducing drugs to cause diabetes. 240 mg kg^−1^ of NT was given 15 min before STZ was administered. 100 mg kg^−1^ of STZ was given twice, on days 0 and 2, after being dissolved in a buffer containing 50 × 10^−3^ m citric acid. Additionally, type 2 diabetes and obesity were created by giving them a 60% fat diet for three weeks. On days 0, 2, and 7 after the induction of diabetes, blood was drawn from a mouse's tail vein. Using a Blood Glucose Test Meter (Accu‐Chek Active Set, Sanmina Ltd., China), plasma glucose concentration was assessed 16 h after the removal of food. After the plasma glucose content was measured, mice were immediately given food. Diabetes was considered to have been induced when fasting blood glucose was 200–300 mg dL^−1^ after a week of combination of drugs and high‐fat diets. The dorsal hairs of the normal and diabetic mice were shaved, and the region was disinfected with povidone‐iodine and alcohol and two full‐thickness wounds were made on the dorsal skin of mice using a sterile 6 mm punch biopsy tool (Kai sterile dermal biopsy punch, kai medical, Japan). After that, the wound was covered with hydrogel dressings made of FSA, DNA@FSA, Si30@FSA, and DNA‐bSi30@FSA. Following surgery, Tegaderm‐film (3 M, USA) was applied to the wounds, and the mice (*n* = 4) were transported in individual cages. Images of the wounds were taken on days 0, 3, 6, 10, and 15, and these images were then processed using Image J software (National Institutes of Health, Bethesda, USA) to determine wound healing ability of 3D‐printed FSA, DNA@FSA, Si30@FSA, and DNA‐bSi30@FSA hydrogel dressing.

### Histological Analysis

The wounds were removed on day 15 following surgery, fixed for 24 h at 4 °C in 4% paraformaldehyde, rinsed with water for at least 4 h, and then embedded in paraffin. Hematoxylin and eosin (H&E) and Masson's trichrome (MT) stains were used in accordance with the manufacturer's instructions to stain the paraffin‐embedded sections of 4 µm thick samples. Using an Olympus BX53 microscope (Olympus Corporation, Shinjuku, Japan), three microscopic fields were taken to measure the cellularity of the H&E‐stained picture and the collagen density of the MT‐stained image. The number of cells per unit area and the collagen density's optical density were calculated using ImageJ software. On 3 mm paraffin slices of the wound skin samples, immunohistochemical (IHC) examination was carried out. Samples were prepared as paraffin blocks, fixed in 10% neutral buffered formalin, and sectioned at a thickness of 3 µm. Using ethyl alcohol with a concentration ranging from 100% to 70%, a deparaffinized sectioned slide was hydrated. After washing in tap water, antigen retrieval is done using the slide that has undergone hydration for 45 min at 90 °C. Use a citrate buffer solution of 0.1 × 10^−3^ m (pH 6.0). Slides should be cleaned twice with distilled water, followed by one additional min of DPBS. Utilize equipment by VENTANA Discovery Ultra automatically (Roche). Using the ImageJ program from the National Institutes of Health, the optical density of TGF‐ *β*, MPO, CD31, and VEGF expression was calculated.

### Statistical Analysis

All assays were performed with a minimum of *n* = 3 per group. Statistical analysis was carried out using IBM SPSS Statistics 26 (IBM, Armonk, NY, USA), and the data are shown as mean ± standard deviation. The one‐way analysis of variance with Tukey's post hoc and Kruskal‐Wallis H‐test with a pairwise comparison was used to determine whether differences were significant. The data were denoted with the following symbols, respectively: (*) for probability less than 0.05 (*p* < 0.05), (**) for *p* < 0.01, (***) for *p* < 0.005, and (****) for *p* < 0.001. All of the statistical significance were noted as Tables S2–S15 (Supporting Information).

## Conflict of Interest

The authors declare no conflict of interest.

## Author Contributions

N.K. and H.L. contributed equally to this work. N.K. and H.L. wrote and edited the paper and contributed to all of the activities. G.H., S.P., Y.N., and S.‐J.B. analyzed chemical structures and mechanical properties. M.K. and M.L. conducted cryoEM analyses. D.E.K. and M.‐J.K. carried out machine learning for determining ink formulation. S.R.S., S.K.O., J.P., T.‐S.J., H.‐E.K., and H.‐D.J. contributed to interpretation and discussion of the experimental results.

## Supporting information

Supporting Information

Supplemental Video 1

## Data Availability

The data that support the findings of this study are available from the corresponding author upon reasonable request.
