# Supplementary material for: 3D‐Printed Functional Hydrogel by DNA‐Induced Biomineralization for Accelerated Diabetic Wound Healing
Source: Adv Sci (Weinh). 2023 Apr 19;10(17):2300816. doi: 10.1002/advs.202300816 (PMC10265106; doi:10.1002/advs.202300816)
Supplement: Supplementary file 1 — Supporting Information [file ADVS-10-2300816-s002.pdf]

ADVANCED SCIENCE
Open Access

Supporting Information

for *Adv. Sci.*, DOI 10.1002/advs.202300816

3D-Printed Functional Hydrogel by DNA-Induced Biomineralization for Accelerated Diabetic Wound Healing

*Nahyun Kim, Hyun Lee, Ginam Han, Minho Kang, Sinwoo Park, Dong Eung Kim, Minyoung Lee, Moon-Jo Kim, Yuhyun Na, SeKwon Oh, Seo-Jun Bang, Tae-Sik Jang, Hyoun-Ee Kim, Jungwon Park, Su Ryon Shin and Hyun-Do Jung**

Supplementary information

3D-printed Functional Hydrogel by DNA-induced Biomineralization for Accelerated Diabetic Wound Healing

Nahyun Kim[1,2,†], Hyun Lee[1,2,†], Ginam Han[1,2], Minho Kang[1,2], Sinwoo Park[1,2], Dong Eung Kim[3], Minyoung Lee[4,5], Moon-Jo Kim[3], Yuhyun Na[1,2], SeKwon Oh[3], Seo-Jun Bang[1,2], Tae-Sik Jang[6], Hyoun-Ee Kim[7], Jungwon Park[4,5], Su Ryon Shin[8], Hyun-Do Jung[1,2,*]

[1] Department of Biomedical-Chemical Engineering, The Catholic University of Korea, Bucheon, 14662, Republic of Korea

[2] Department of Biotechnology, The Catholic University of Korea, Bucheon, 14662, Republic of Korea

[3] Research Institute of Advanced Manufacturing & Materials Technology, Korea Institute of Industrial Technology, Incheon, 21999, Republic of Korea

[4] School of Chemical and Biological Engineering, and Institute of Chemical Processes (ICP), Seoul National University, Seoul, 08826, Republic of Korea

[5] Center for Nanoparticle Research, Institute of Basic Science (IBS), Seoul, 08826, Republic of Korea

[6] Department of Materials Science and Engineering, Chosun University, Gwangju, 61452, Republic of Korea

[7] Department of Materials Science and Engineering, Seoul National University, Seoul, 08826, Republic of Korea

[8] Division of Engineering in Medicine, Department of Medicine, Harvard Medical School, and Brigham and Women's Hospital, Cambridge, Massachusetts 02139, United States

[†]Both authors contributed equally to this work.

---

[*] Corresponding author. Tel.: +82-2-2164-4357

E-mail address: hdjung@catholic.ac.kr (Hyun-Do Jung)

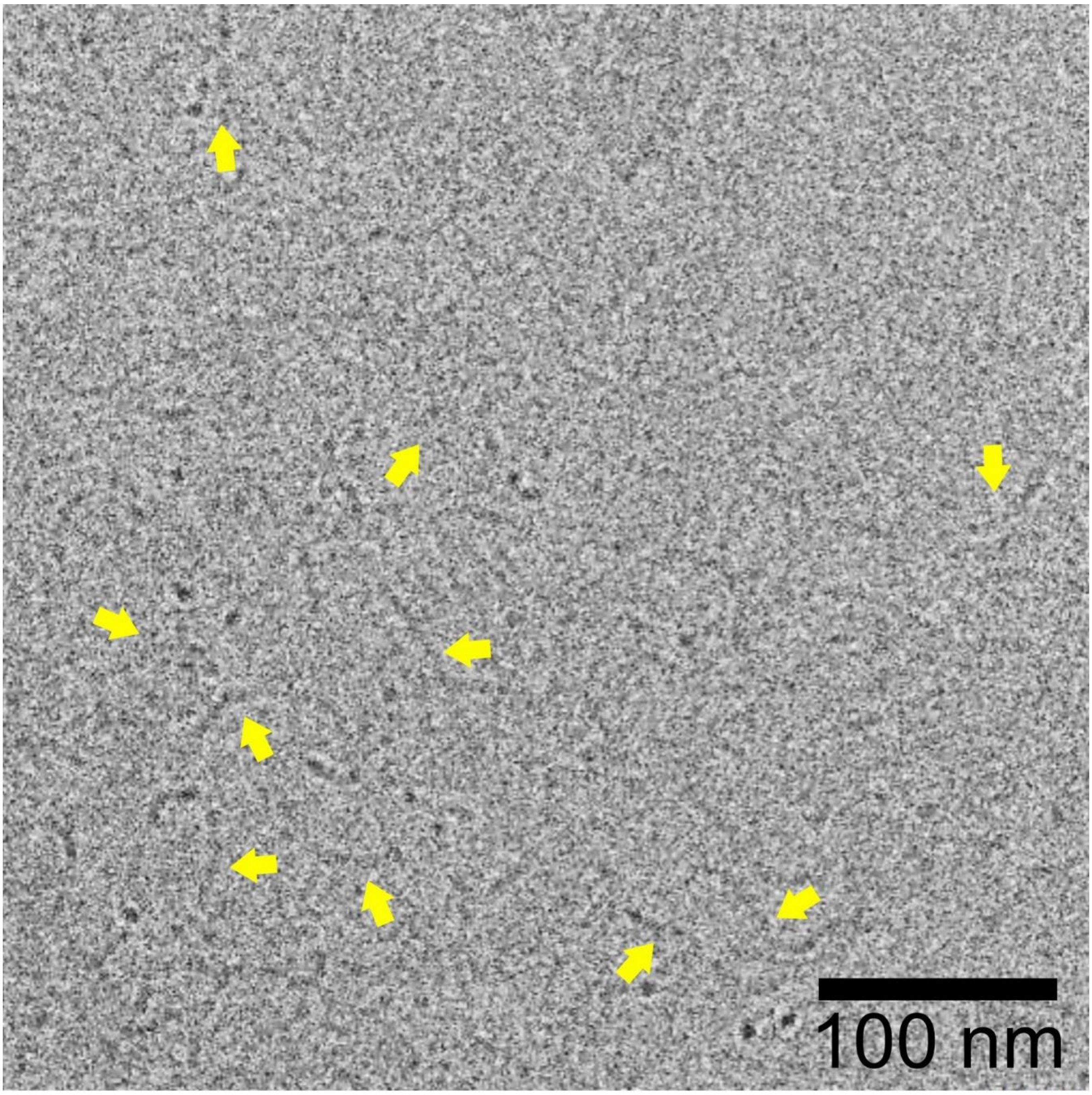

Figure S1. Cryo-TEM image of DNA (Yellow arrows directing DNA strand).

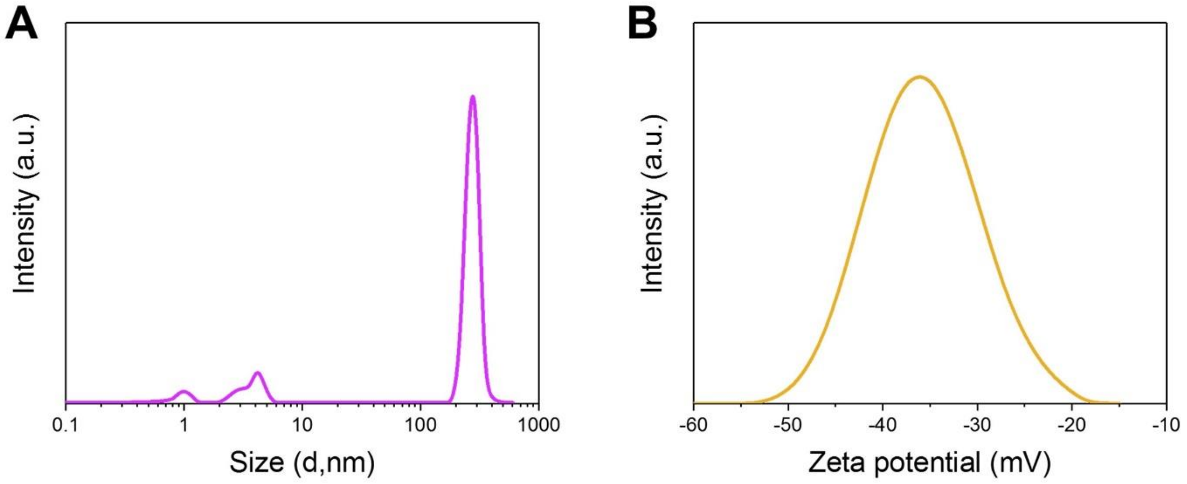

Figure S**2**. (A) Size distribution of DNA measured by DLS analysis. (B) Zeta potential distribution of incorporated DNA at pH 10.5.

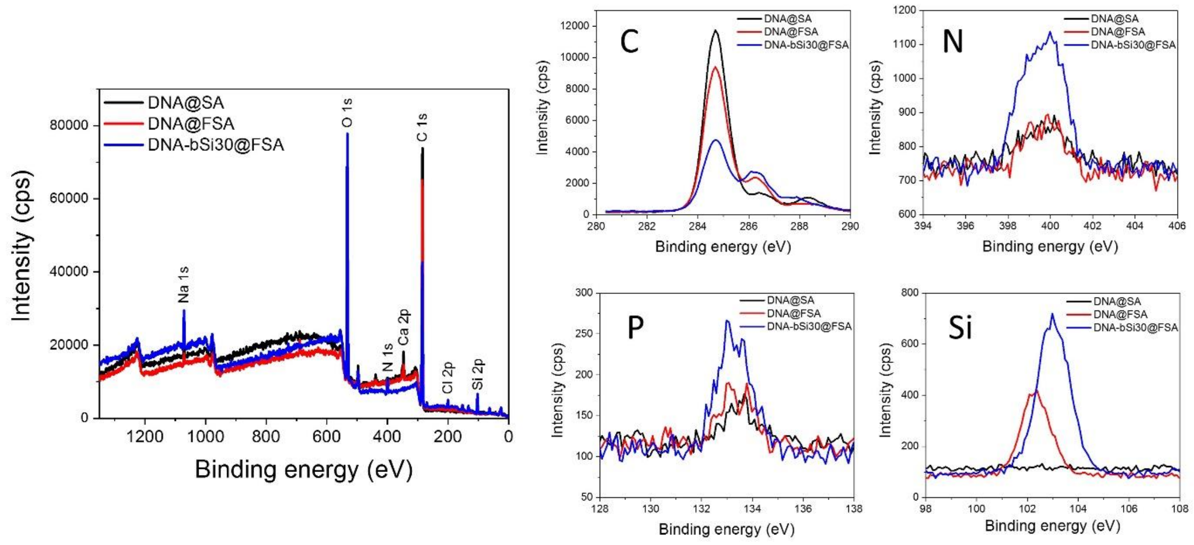

Figure S3. XPS results of DNA@SA, DNA@FSA, and DNA-bSi30@FSA at different elements.

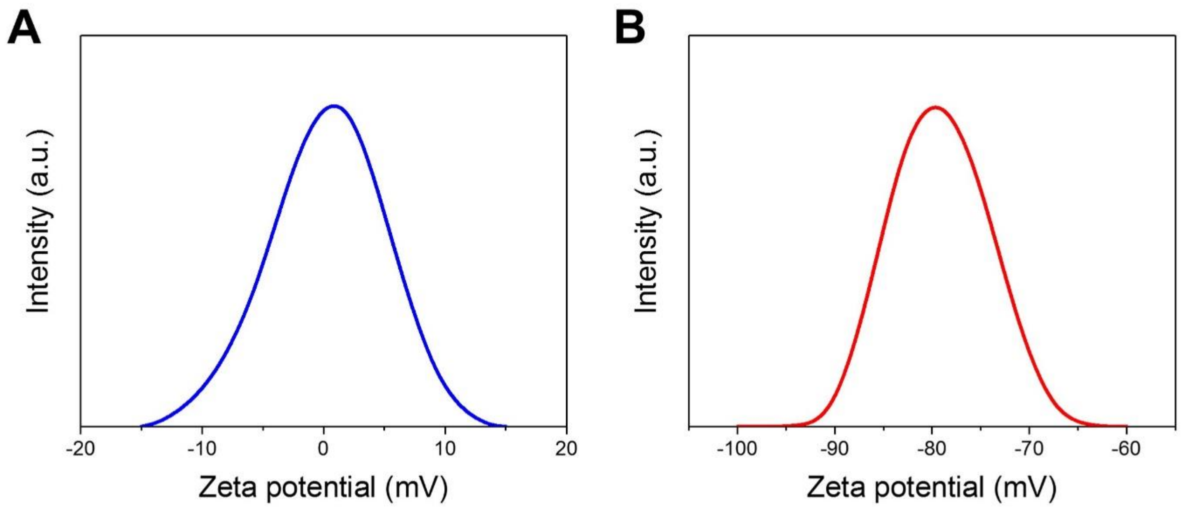

Figure S4. Zeta potential distributions of (A) silica precursor and (B) DNA-bSi@FSA ink.

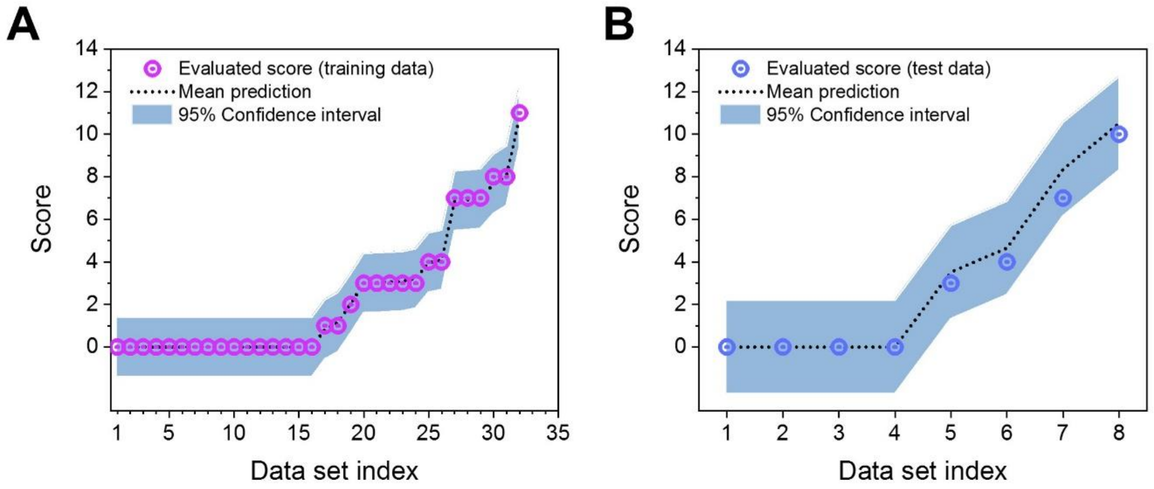

Figure S5. Evaluated score (circle symbol) and predicted mean score (dot line) with the 95% confidence interval (blue area) based on (A) 32 training data sets and (B) 8 test data sets.

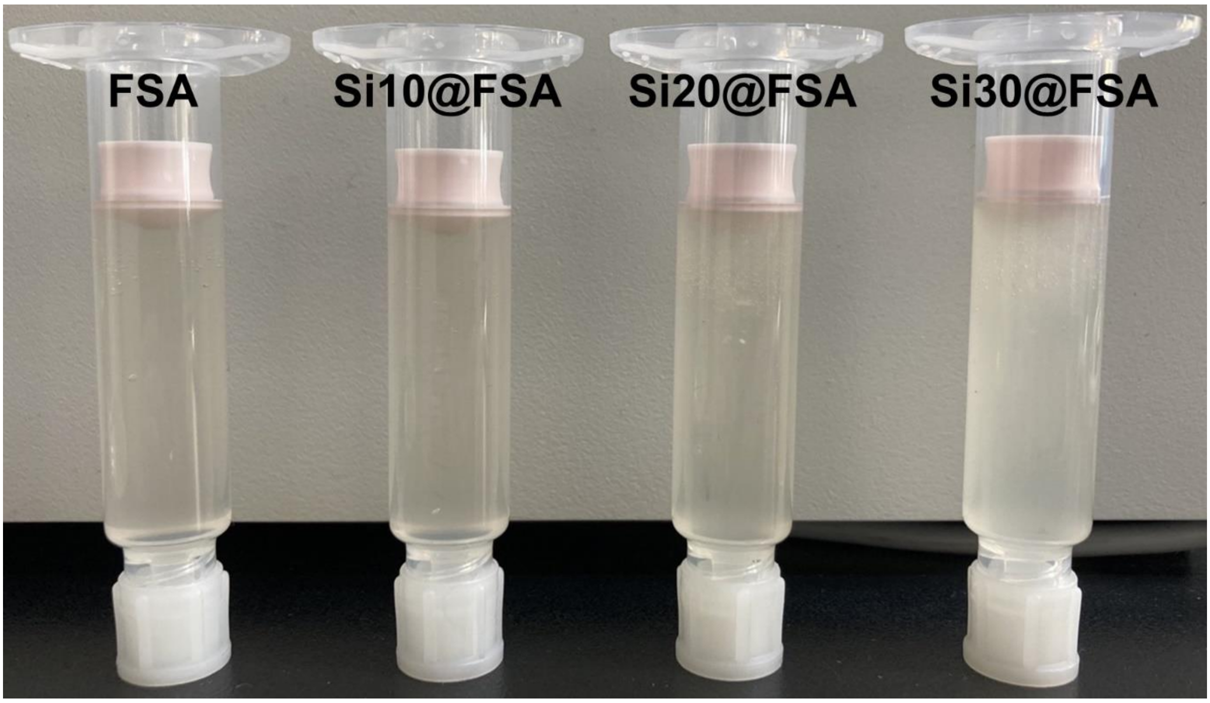

Figure S6. Optical images of hydrogel inks with different silica contents.

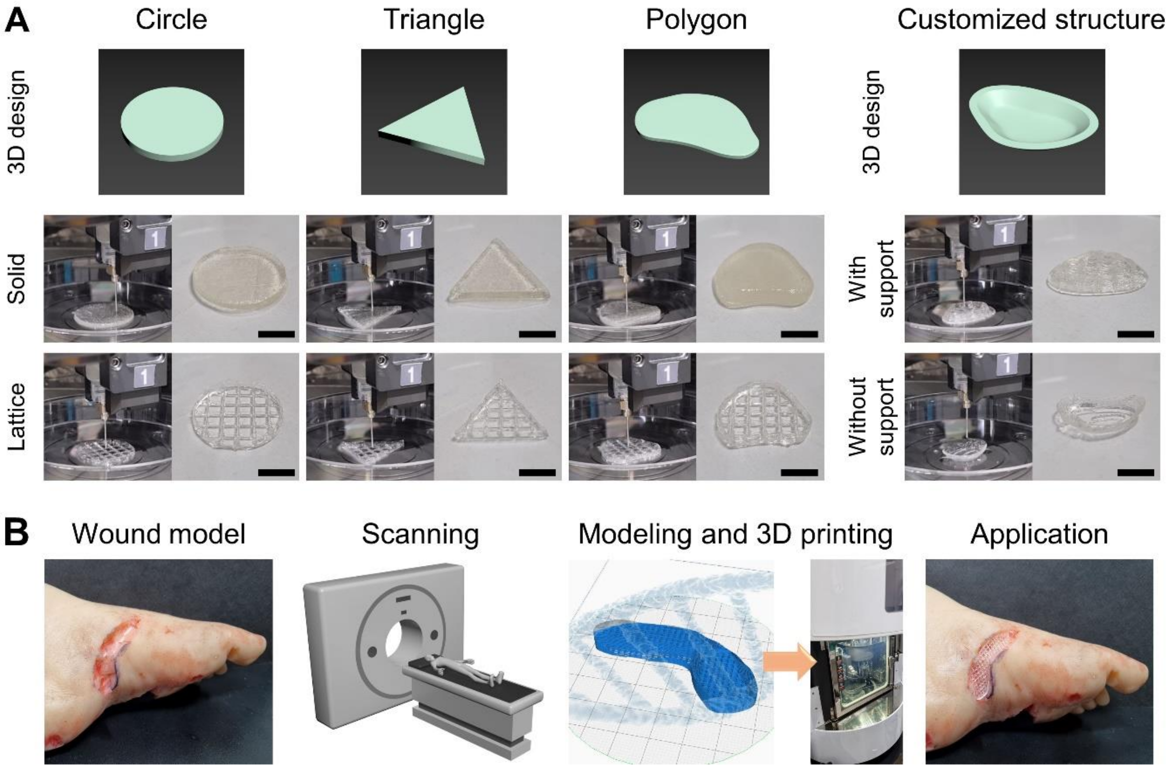

Figure S7. (A) 3D printing of DNA-bSi@FSA hydrogel dressings with circular, triangular, polygonal, and customized structure (scale bar: 10 mm). (B) Fabrication of 3D-printed DNA-bSi@FSA hydrogel dressing for a sample clinical application.

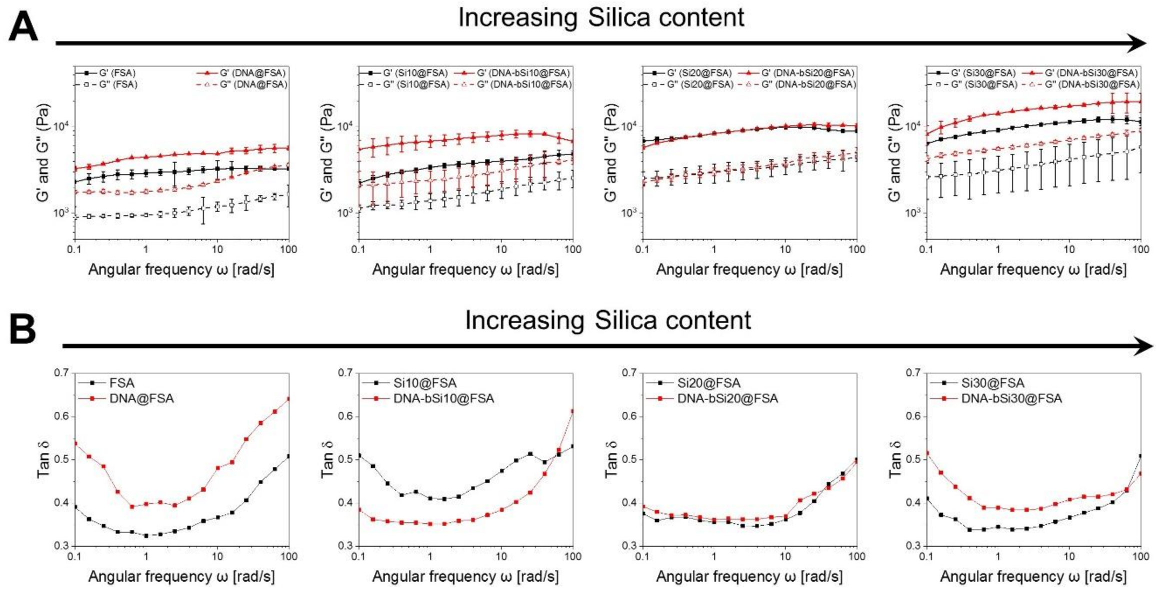

Figure S8. (A) Storage and loss modulus of fabricated hydrogel dressing and (B) computed tan δ following angular frequency.

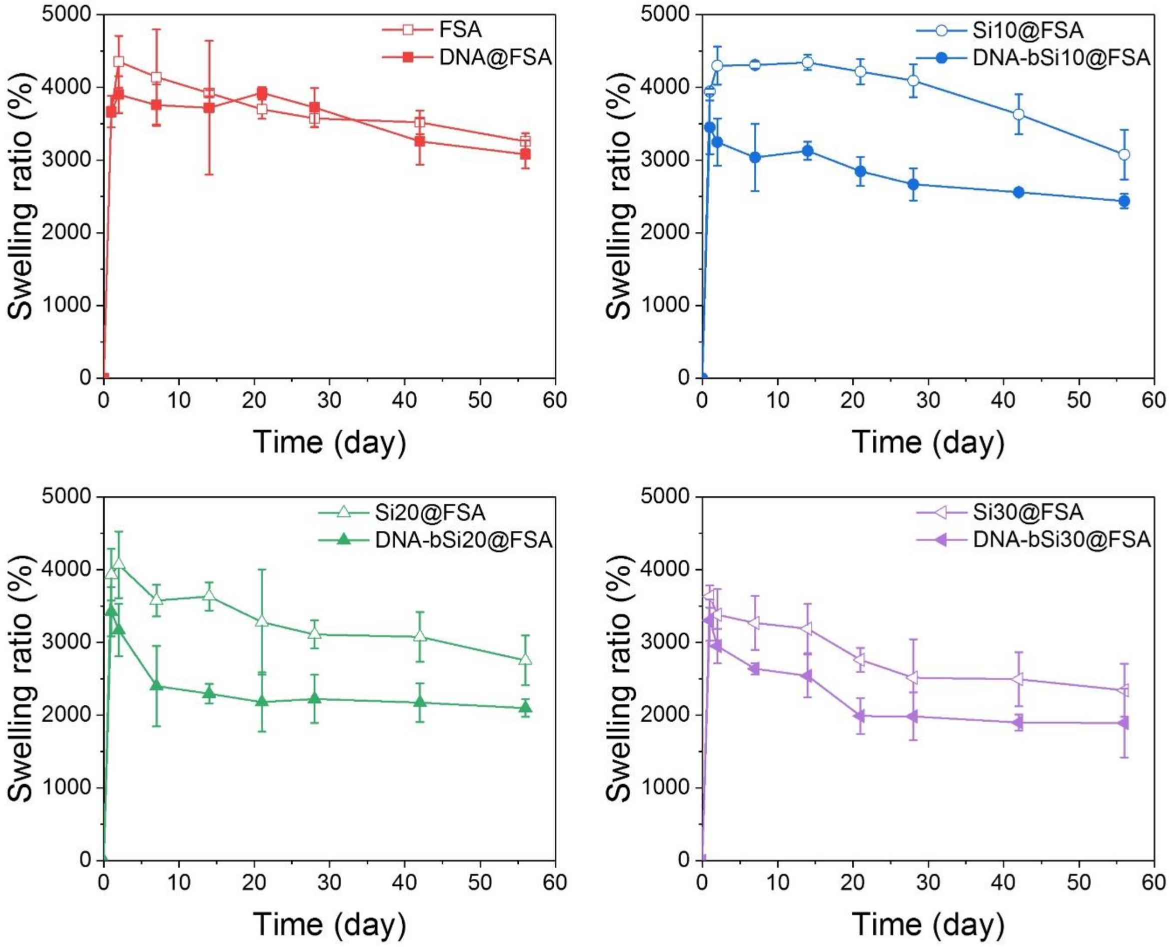

Figure S9. Monitored swelling ratio of 3D-printed hydrogel dressings.

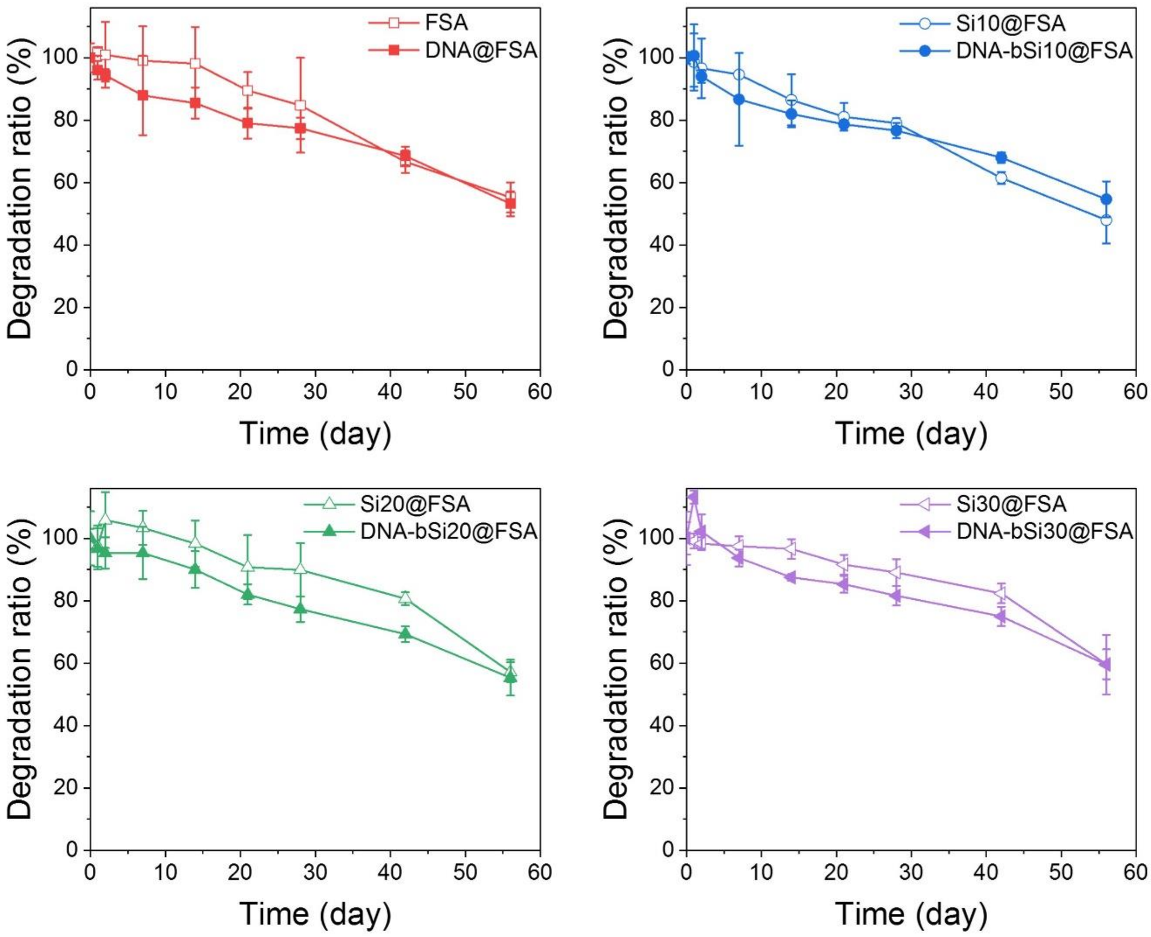

Figure S10. Degradation ratio of 3D-printed hydrogel dressings in DPBS.

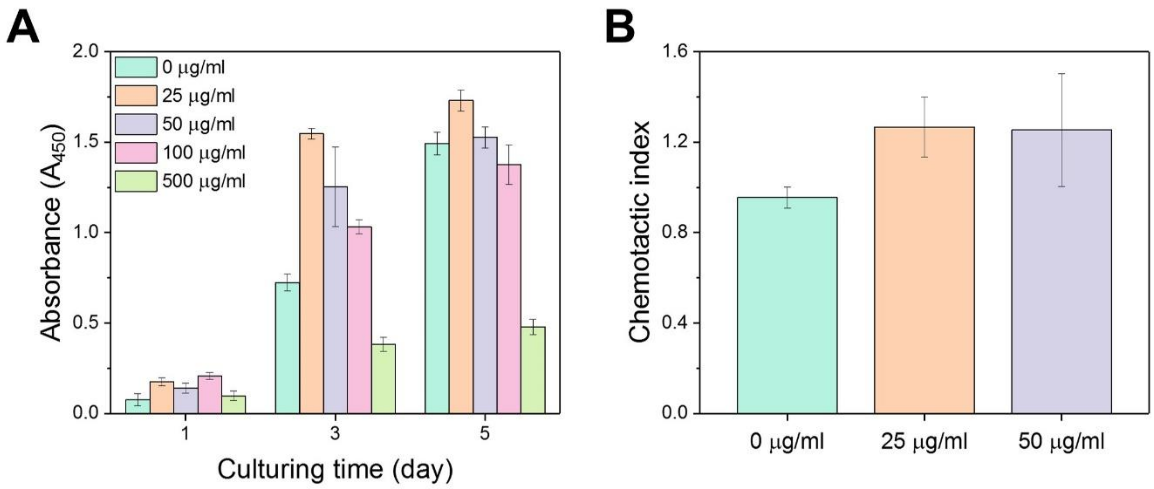

Figure S11. Comparison of (A) L929 proliferation and (B) chemotactic index with altered DNA concentration.

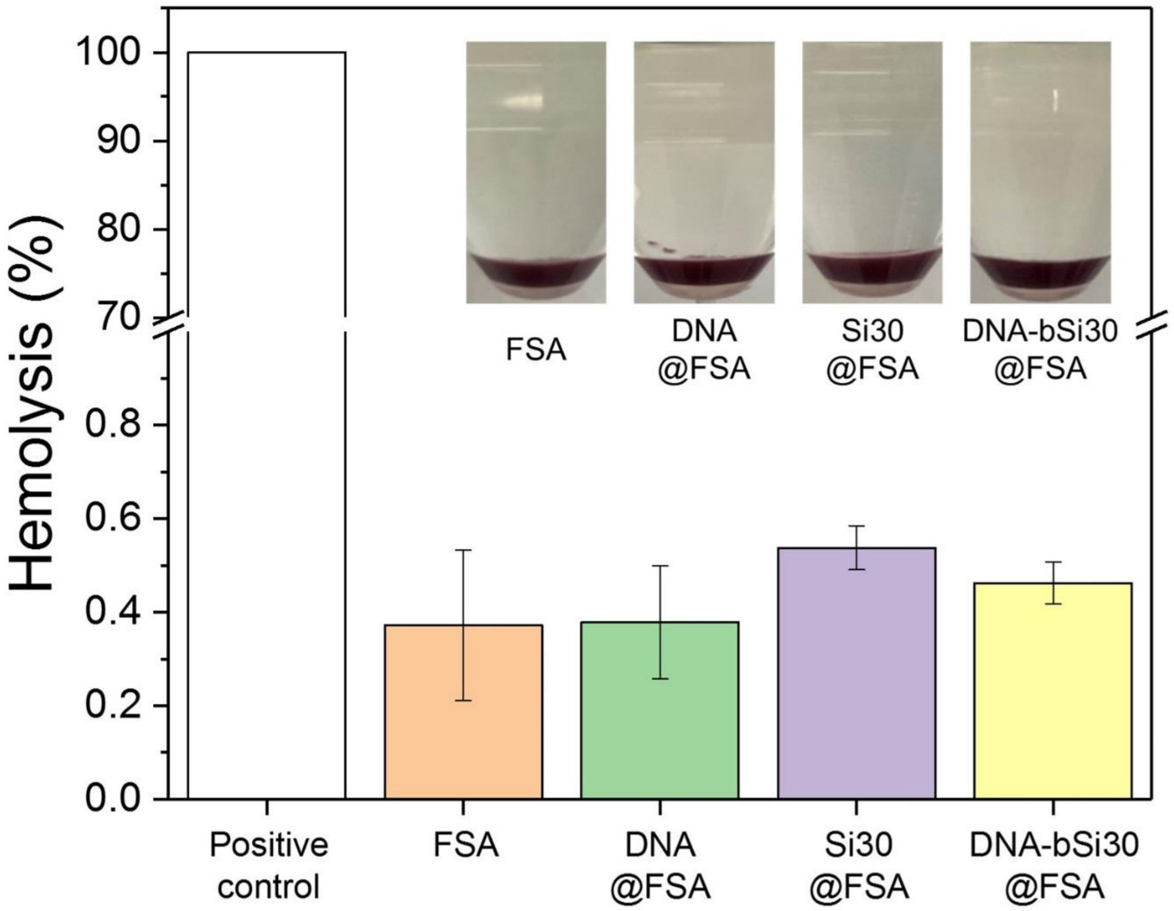

Figure S12. Hemolysis rates of FSA, DNA@ FSA, Si30@ FSA, and DNA-bSi30@ FSA (Inset represents image of samples after centrifugation).

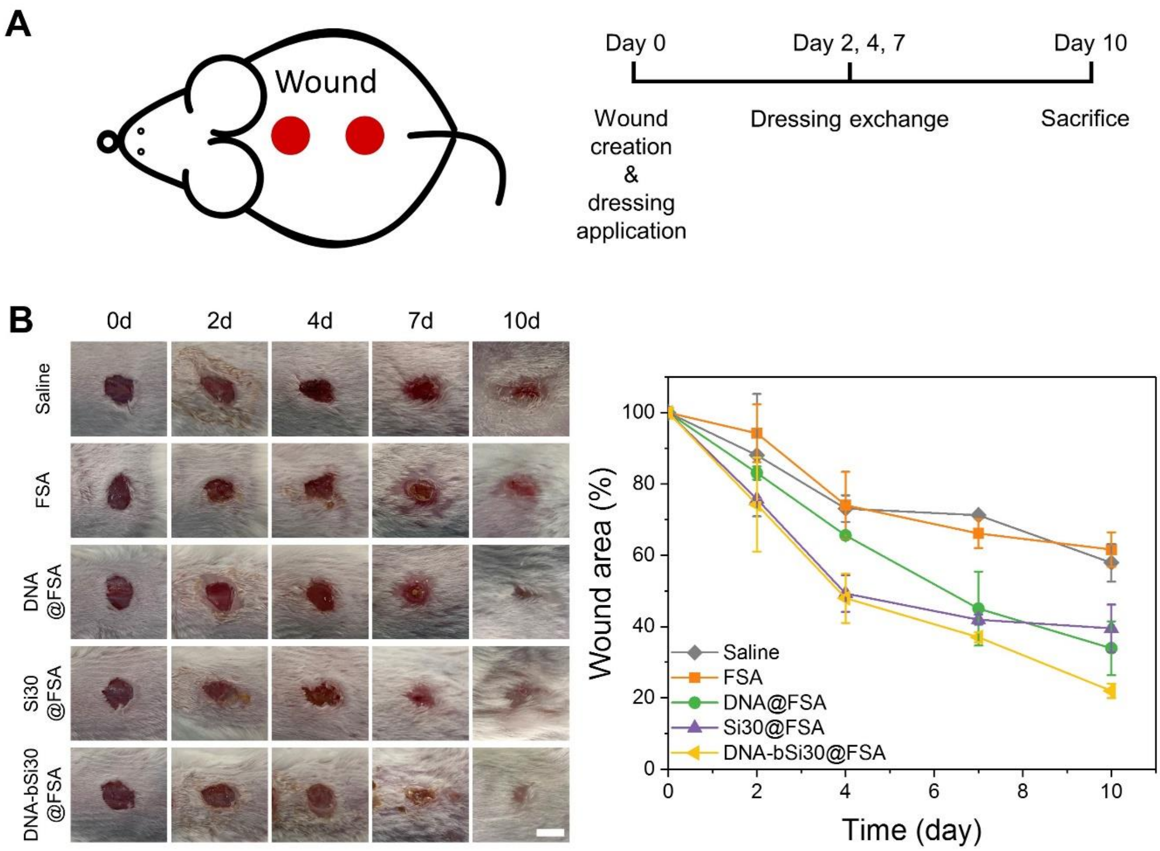

Figure S13. (A) Scheme and the time table of *in vivo* acute wound healing experiments. (B) Optical images of wound sites and computed wound area at 0, 2, 4, 7, and 10 days (scale bar: 5 mm).

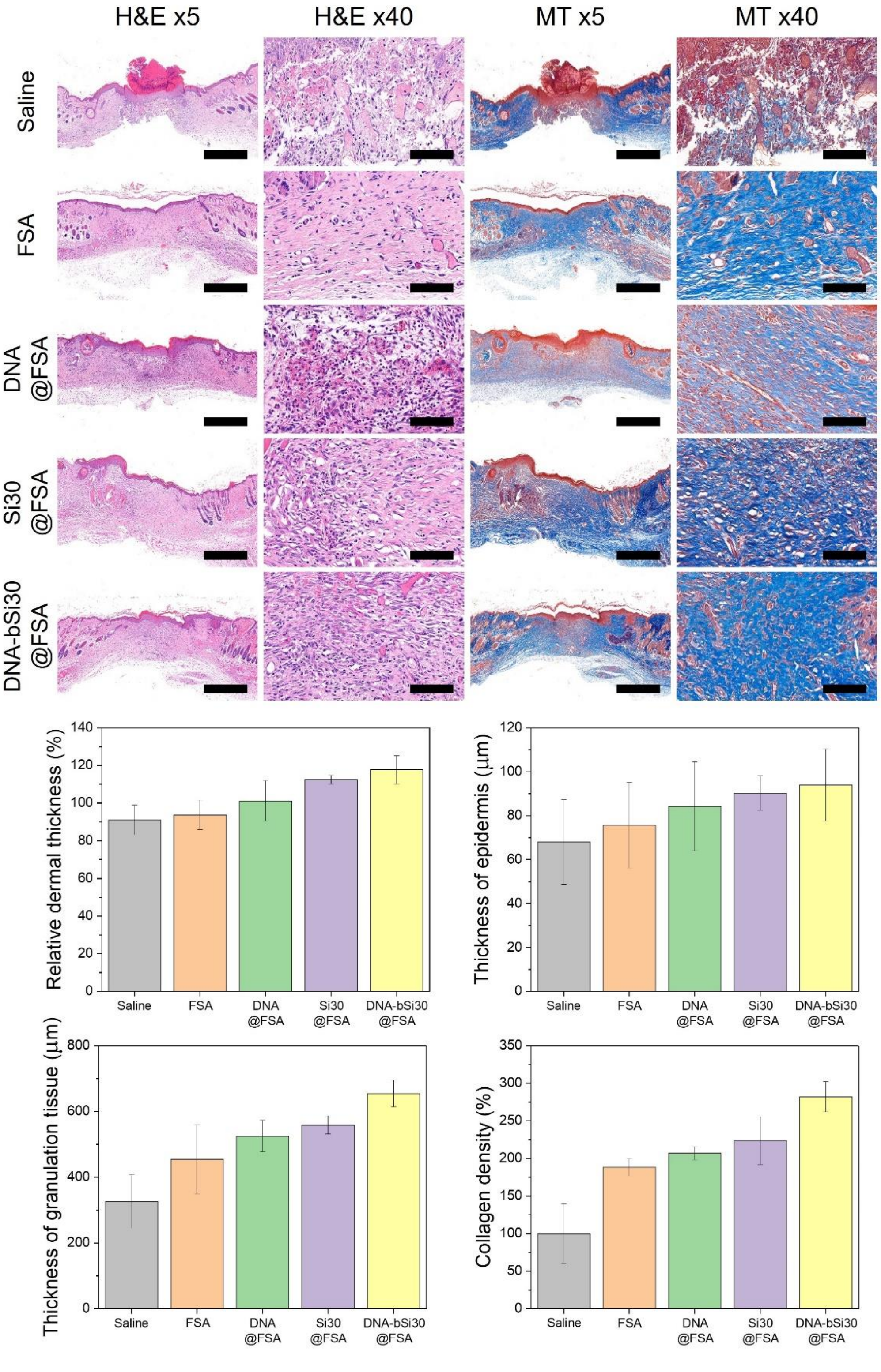

Figure S14. Images of H&E and MT stained skin tissues and measured factors related with skin tissue regeneration of acute *in vivo* wound healing experiment (scale bar : 500 μm for x5 magnification and 50 μm for x40 magnification).

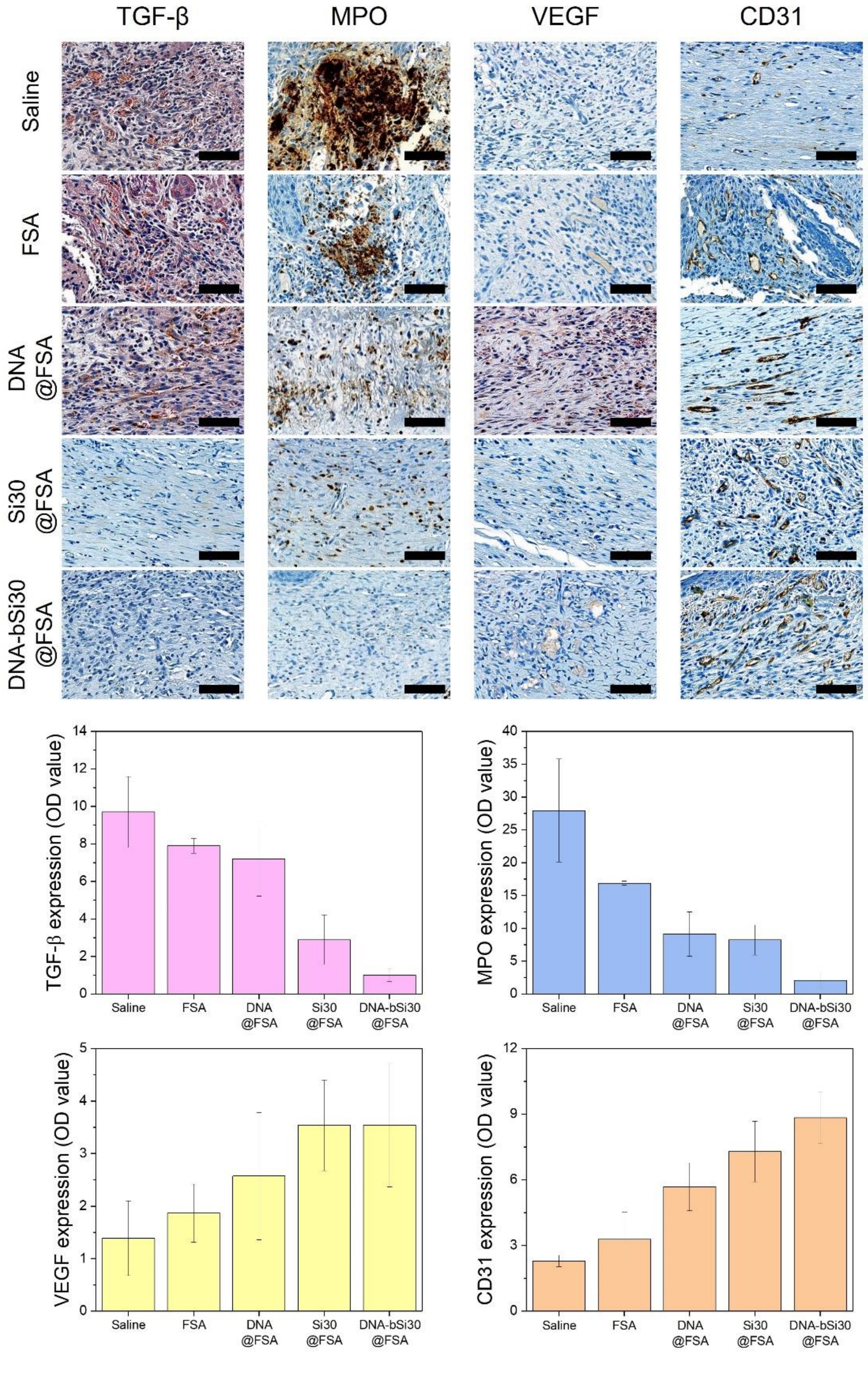

Figure S15. Immunohistochemical analysis regarding inflammation (TGF-β and MPO) and angiogenesis (VEGF and CD31) of skin tissues of acute *in vivo* wound healing experiment (scale bar: 50 μm).

Table S1. Dataset for machine learning modeling with input variables (FSA concentration, nozzle size, temperature, pneumatic pressure) and output variable (score).

Input variable				Output variable
FSA concentration (% w/v)	Nozzle size (mm)	Temperature (℃)	Pneumatic pressure (kPa)	Score
1	0.2	27	20	0
			60	0
		37	20	0
			60	0
	0.4	27	20	3
			60	3
		37	20	7
			60	7
2	0.2	27	20	0
			60	0
		37	20	0
			60	0
	0.4	27	20	3
			60	4
		37	20	7
			60	8
2.5	0.2	27	20	0
			60	0
		37	20	0
			60	0
	0.4	27	20	3
			60	4
		37	20	7
			60	10
3	0.2	27	20	0
			60	0
		37	20	0
			60	0
	0.4	27	20	3
			60	4
		37	20	7
			60	12
4	0.2	27	20	0
			60	0
		37	20	0
			60	0
	0.4	27	20	1
			60	1
		37	20	2
			60	3

Table S2. Intergroup statistical comparisons of storage modulus and loss modulus of ink at 1Hz.

Sample 1 - Sample 2	Significance	
	Storage modulus	Loss modulus
FSA - Si10@FSA	1.000	1.000
FSA - Si20@FSA	0.005**	0.005**
FSA - Si30@FSA	0.000****	0.000****
FSA - DNA@FSA	0.952	0.952
FSA - DNA-bSi10@FSA	0.269	0.269
FSA - DNA-bSi20@FSA	0.004***	0.004***
FSA - DNA-bSi30@FSA	0.000****	0.000****
Si10@FSA - Si20@FSA	0.009**	0.009**
Si10@FSA - Si30@FSA	0.000****	0.000****
Si10@FSA - DNA@FSA	0.994	0.994
Si10@FSA - DNA-bSi10@FSA	0.433	0.433
Si10@FSA - DNA-bSi20@FSA	0.008**	0.008**
Si10@FSA - DNA-bSi30@FSA	0.000****	0.000****
Si20@FSA - Si30@FSA	0.386	0.386
Si20@FSA - DNA@FSA	0.039*	0.039*
Si20@FSA - DNA-bSi10@FSA	0.403	0.403
Si20@FSA - DNA-bSi20@FSA	1.000	1.000
Si20@FSA - DNA-bSi30@FSA	0.000****	0.000****
Si30@FSA - DNA@FSA	0.001***	0.001***
Si30@FSA - DNA-bSi10@FSA	0.008**	0.008**
Si30@FSA - DNA-bSi20@FSA	0.430	0.430
Si30@FSA - DNA-bSi30@FSA	0.002***	0.002***
DNA@FSA - DNA-bSi10@FSA	0.844	0.844
DNA@FSA - DNA-bSi20@FSA	0.033*	0.033*
DNA@FSA - DNA-bSi30@FSA	0.000****	0.000****
DNA-bSi10@FSA - DNA-bSi20@FSA	0.361	0.361
DNA-bSi10@FSA - DNA-bSi30@FSA	0.000****	0.000****
DNA-bSi20@FSA - DNA-bSi30@FSA	0.000****	0.000****

Table S3. Intergroup statistical comparisons of average pore size.

Sample 1 - Sample 2	Significance
FSA - Si10@FSA	0.432
FSA - Si20@FSA	0.391
FSA - Si30@FSA	0.115
FSA - DNA@FSA	0.389
FSA - DNA-bSi10@FSA	0.341
FSA - DNA-bSi20@FSA	0.326
FSA - DNA-bSi30@FSA	0.004***
Si10@FSA - Si20@FSA	0.918
Si10@FSA - Si30@FSA	0.374
Si10@FSA - DNA@FSA	0.094
Si10@FSA - DNA-bSi10@FSA	0.869
Si10@FSA - DNA-bSi20@FSA	0.827
Si10@FSA - DNA-bSi30@FSA	0.028*
Si20@FSA - Si30@FSA	0.444
Si20@FSA - DNA@FSA	0.086
Si20@FSA - DNA-bSi10@FSA	0.957
Si20@FSA - DNA-bSi20@FSA	0.912
Si20@FSA - DNA-bSi30@FSA	0.046*
Si30@FSA - DNA@FSA	0.017*
Si30@FSA - DNA-bSi10@FSA	0.453
Si30@FSA - DNA-bSi20@FSA	0.500
Si30@FSA - DNA-bSi30@FSA	0.303
DNA@FSA - DNA-bSi10@FSA	0.065
DNA@FSA - DNA-bSi20@FSA	0.064
DNA@FSA - DNA-bSi30@FSA	0.000****
DNA-bSi10@FSA - DNA-bSi20@FSA	0.952
DNA-bSi10@FSA - DNA-bSi30@FSA	0.055
DNA-bSi20@FSA - DNA-bSi30@FSA	0.039*

Table S4. Intergroup statistical comparisons of storage modulus and loss modulus of scaffolds at 1Hz.

Sample 1 - Sample 2	Significance	
	Storage modulus	Loss modulus
FSA - Si10@FSA	0.603	0.957
FSA - Si20@FSA	0.018*	0.004***
FSA - Si30@FSA	0.002***	0.000****
FSA - DNA@FSA	0.299	0.536
FSA - DNA-bSi10@FSA	0.119	0.053
FSA - DNA-bSi20@FSA	0.021*	0.003***
FSA - DNA-bSi30@FSA	0.000****	0.000****
Si10@FSA - Si20@FSA	0.065	0.029*
Si10@FSA - Si30@FSA	0.009**	0.000****
Si10@FSA - DNA@FSA	0.603	0.982
Si10@FSA - DNA-bSi10@FSA	0.299	0.315
Si10@FSA - DNA-bSi20@FSA	0.073	0.024*
Si10@FSA - DNA-bSi30@FSA	0.002***	0.000****
Si20@FSA - Si30@FSA	0.453	0.205
Si20@FSA - DNA@FSA	0.184	0.149
Si20@FSA - DNA-bSi10@FSA	0.419	0.854
Si20@FSA - DNA-bSi20@FSA	0.954	1.000
Si20@FSA - DNA-bSi30@FSA	0.204	0.000****
Si30@FSA - DNA@FSA	0.038*	0.001***
Si30@FSA - DNA-bSi10@FSA	0.119	0.017*
Si30@FSA - DNA-bSi20@FSA	0.419	0.238
Si30@FSA - DNA-bSi30@FSA	0.603	0.052
DNA@FSA - DNA-bSi10@FSA	0.603	0.809
DNA@FSA - DNA-bSi20@FSA	0.204	0.126
DNA@FSA - DNA-bSi30@FSA	0.009**	0.000****
DNA-bSi10@FSA - DNA-bSi20@FSA	0.453	0.810
DNA-bSi10@FSA - DNA-bSi30@FSA	0.038*	0.000****
DNA-bSi20@FSA - DNA-bSi30@FSA	0.184	0.000****

Table S5. Intergroup statistical comparisons of compressive properties of fabricated 3D-printed hydrogel dressings.

Sample 1 - Sample 2	Significance	
	Compressive strength	Compressive modulus
FSA - Si10@FSA	0.488	Not statistically different between the groups
FSA - Si20@FSA	0.073	
FSA - Si30@FSA	0.028*	
FSA - DNA@FSA	0.488	
FSA - DNA-bSi10@FSA	0.204	
FSA - DNA-bSi20@FSA	0.018*	
FSA - DNA-bSi30@FSA	0.001***	
Si10@FSA - Si20@FSA	0.273	
Si10@FSA - Si30@FSA	0.133	
Si10@FSA - DNA@FSA	1.000	
Si10@FSA - DNA-bSi10@FSA	0.564	
Si10@FSA - DNA-bSi20@FSA	0.094	
Si10@FSA - DNA-bSi30@FSA	0.011*	
Si20@FSA - Si30@FSA	0.686	
Si20@FSA - DNA@FSA	0.273	
Si20@FSA - DNA-bSi10@FSA	0.603	
Si20@FSA - DNA-bSi20@FSA	0.564	
Si20@FSA - DNA-bSi30@FSA	0.149	
Si30@FSA - DNA@FSA	0.133	
Si30@FSA - DNA-bSi10@FSA	0.356	
Si30@FSA - DNA-bSi20@FSA	0.862	
Si30@FSA - DNA-bSi30@FSA	0.299	
DNA@FSA - DNA-bSi10@FSA	0.564	
DNA@FSA - DNA-bSi20@FSA	0.094	
DNA@FSA - DNA-bSi30@FSA	0.011*	
DNA-bSi10@FSA - DNA-bSi20@FSA	0.273	
DNA-bSi10@FSA - DNA-bSi30@FSA	0.050	
DNA-bSi20@FSA - DNA-bSi30@FSA	0.386	

Table S6. Intergroup statistical comparisons of fluorescent intensity.

Sample 1 - Sample 2	Significance
FSA - Si10@FSA	1.000
FSA - Si20@FSA	0.995
FSA - Si30@FSA	0.780
FSA - DNA@FSA	1.000
FSA - DNA-bSi10@FSA	0.999
FSA - DNA-bSi20@FSA	0.818
FSA - DNA-bSi30@FSA	0.035*
Si10@FSA - Si20@FSA	1.000
Si10@FSA - Si30@FSA	0.949
Si10@FSA - DNA@FSA	1.000
Si10@FSA - DNA-bSi10@FSA	1.000
Si10@FSA - DNA-bSi20@FSA	0.964
Si10@FSA - DNA-bSi30@FSA	0.088
Si20@FSA - Si30@FSA	0.992
Si20@FSA - DNA@FSA	1.000
Si20@FSA - DNA-bSi10@FSA	1.000
Si20@FSA - DNA-bSi20@FSA	0.995
Si20@FSA - DNA-bSi30@FSA	0.160
Si30@FSA - DNA@FSA	0.992
Si30@FSA - DNA-bSi10@FSA	0.967
Si30@FSA - DNA-bSi20@FSA	1.000
Si30@FSA - DNA-bSi30@FSA	0.540
DNA@FSA - DNA-bSi10@FSA	1.000
DNA@FSA - DNA-bSi20@FSA	0.943
DNA@FSA - DNA-bSi30@FSA	0.071
DNA-bSi10@FSA - DNA-bSi20@FSA	0.978
DNA-bSi10@FSA - DNA-bSi30@FSA	0.104
DNA-bSi20@FSA - DNA-bSi30@FSA	0.495

Table S7. Intergroup statistical comparisons of cell proliferation cultured on the scaffolds after 3 and 5 days.

Sample 1 - Sample 2	Significance	
	3d	5d
FSA - Si10@FSA	0.908	0.817
FSA - Si20@FSA	0.273	0.149
FSA - Si30@FSA	0.024*	0.028*
FSA - DNA@FSA	0.326	0.862
FSA - DNA-bSi10@FSA	0.043*	0.299
FSA - DNA-bSi20@FSA	0.005**	0.038*
FSA - DNA-bSi30@FSA	0.001***	0.006**
Si10@FSA - Si20@FSA	0.326	0.094
Si10@FSA - Si30@FSA	0.033*	0.015*
Si10@FSA - DNA@FSA	0.386	0.686
Si10@FSA - DNA-bSi10@FSA	0.057	0.204
Si10@FSA - DNA-bSi20@FSA	0.007**	0.021*
Si10@FSA - DNA-bSi30@FSA	0.001***	0.003***
Si20@FSA - Si30@FSA	0.248	0.453
Si20@FSA - DNA@FSA	0.908	0.204
Si20@FSA - DNA-bSi10@FSA	0.356	0.686
Si20@FSA - DNA-bSi20@FSA	0.083	0.525
Si20@FSA - DNA-bSi30@FSA	0.021*	0.184
Si30@FSA - DNA@FSA	0.204	0.043*
Si30@FSA - DNA-bSi10@FSA	0.817	0.248
Si30@FSA - DNA-bSi20@FSA	0.564	0.908
Si30@FSA - DNA-bSi30@FSA	0.248	0.564
DNA@FSA - DNA-bSi10@FSA	0.299	0.386
DNA@FSA - DNA-bSi20@FSA	0.065	0.057
DNA@FSA - DNA-bSi30@FSA	0.015*	0.009**
DNA-bSi10@FSA - DNA-bSi20@FSA	0.419	0.299
DNA-bSi10@FSA - DNA-bSi30@FSA	0.166	0.083
DNA-bSi20@FSA - DNA-bSi30@FSA	0.564	0.488

Table S8. Intergroup statistical comparisons of cell migration.

Sample 1 - Sample 2	Significance	Sample 1 - Sample 2	Significance
FSA_6h - Si10@FSA_6h	1.000	DNA@FSA_6h - Si30@FSA_12h	0.189
FSA_6h - Si20@FSA_6h	0.268	DNA@FSA_6h - DNA@FSA_12h	0.221
FSA_6h - Si30@FSA_6h	0.307	DNA@FSA_6h - DNA-bSi10@FSA_12h	0.210
FSA_6h - DNA@FSA_6h	0.351	DNA@FSA_6h - DNA-bSi20@FSA_12h	0.027*
FSA_6h - DNA-bSi10@FSA_6h	0.243	DNA@FSA_6h - DNA-bSi30@FSA_12h	0.006**
FSA_6h - DNA-bSi20@FSA_6h	0.041*	DNA-bSi10@FSA_6h - DNA-bSi20@FSA_6h	0.382
FSA_6h - DNA-bSi30@FSA_6h	0.001***	DNA-bSi10@FSA_6h - DNA-bSi30@FSA_6h	0.044*
FSA_6h - FSA_12h	0.085	DNA-bSi10@FSA_6h - FSA_12h	0.580
FSA_6h - Si10@FSA_12h	0.058	DNA-bSi10@FSA_6h - Si10@FSA_12h	0.466
FSA_6h - Si20@FSA_12h	0.029*	DNA-bSi10@FSA_6h - Si20@FSA_12h	0.307
FSA_6h - Si30@FSA_12h	0.025*	DNA-bSi10@FSA_6h - Si30@FSA_12h	0.281
FSA_6h - DNA@FSA_12h	0.031*	DNA-bSi10@FSA_6h - DNA@FSA_12h	0.321
FSA_6h - DNA-bSi10@FSA_12h	0.029*	DNA-bSi10@FSA_6h - DNA-bSi10@FSA_12h	0.307
FSA_6h - DNA-bSi20@FSA_12h	0.002***	DNA-bSi10@FSA_6h - DNA-bSi20@FSA_12h	0.047*
FSA_6h - DNA-bSi30@FSA_12h	0.000****	DNA-bSi10@FSA_6h - DNA-bSi30@FSA_12h	0.011*
Si10@FSA_6h - Si20@FSA_6h	0.268	DNA-bSi20@FSA_6h - DNA-bSi30@FSA_6h	0.255
Si10@FSA_6h - Si30@FSA_6h	0.307	DNA-bSi20@FSA_6h - FSA_12h	0.748
Si10@FSA_6h - DNA@FSA_6h	0.351	DNA-bSi20@FSA_6h - Si10@FSA_12h	0.884
Si10@FSA_6h - DNA-bSi10@FSA_6h	0.243	DNA-bSi20@FSA_6h - Si20@FSA_12h	0.884
Si10@FSA_6h - DNA-bSi20@FSA_6h	0.041*	DNA-bSi20@FSA_6h - Si30@FSA_12h	0.838
Si10@FSA_6h - DNA-bSi30@FSA_6h	0.001***	DNA-bSi20@FSA_6h - DNA@FSA_12h	0.907
Si10@FSA_6h - FSA_12h	0.085	DNA-bSi20@FSA_6h - DNA-bSi10@FSA_12h	0.884
Si10@FSA_6h - Si10@FSA_12h	0.058	DNA-bSi20@FSA_6h - DNA-bSi20@FSA_12h	0.268
Si10@FSA_6h - Si20@FSA_12h	0.029*	DNA-bSi20@FSA_6h - DNA-bSi30@FSA_12h	0.096
Si10@FSA_6h - Si30@FSA_12h	0.025*	DNA-bSi30@FSA_6h - FSA_12h	0.145
Si10@FSA_6h - DNA@FSA_12h	0.031*	DNA-bSi30@FSA_6h - Si10@FSA_12h	0.199
Si10@FSA_6h - DNA-bSi10@FSA_12h	0.029*	DNA-bSi30@FSA_6h - Si20@FSA_12h	0.321
Si10@FSA_6h - DNA-bSi20@FSA_12h	0.002***	DNA-bSi30@FSA_6h - Si30@FSA_12h	0.351
Si10@FSA_6h - DNA-bSi30@FSA_12h	0.000****	DNA-bSi30@FSA_6h - DNA@FSA_12h	0.307
Si20@FSA_6h - Si30@FSA_6h	0.930	DNA-bSi30@FSA_6h - DNA-bSi10@FSA_12h	0.321
Si20@FSA_6h - DNA@FSA_6h	0.861	DNA-bSi30@FSA_6h - DNA-bSi20@FSA_12h	0.977
Si20@FSA_6h - DNA-bSi10@FSA_6h	0.953	DNA-bSi30@FSA_6h - DNA-bSi30@FSA_12h	0.600
Si20@FSA_6h - DNA-bSi20@FSA_6h	0.351	FSA_12h - Si10@FSA_12h	0.793
Si20@FSA_6h - DNA-bSi30@FSA_6h	0.038*	FSA_12h - Si20@FSA_12h	0.641
Si20@FSA_6h - FSA_12h	0.540	FSA_12h - Si30@FSA_12h	0.600
Si20@FSA_6h - Si10@FSA_12h	0.431	FSA_12h - DNA@FSA_12h	0.662
Si20@FSA_6h - Si20@FSA_12h	0.281	FSA_12h - DNA-bSi10@FSA_12h	0.641
Si20@FSA_6h - Si30@FSA_12h	0.255	FSA_12h - DNA-bSi20@FSA_12h	0.153
Si20@FSA_6h - DNA@FSA_12h	0.294	FSA_12h - DNA-bSi30@FSA_12h	0.047*
Si20@FSA_6h - DNA-bSi10@FSA_12h	0.281	Si10@FSA_12h - Si20@FSA_12h	0.771
Si20@FSA_6h - DNA-bSi20@FSA_12h	0.041*	Si10@FSA_12h - Si30@FSA_12h	0.726
Si20@FSA_6h - DNA-bSi30@FSA_12h	0.009**	Si10@FSA_12h - DNA@FSA_12h	0.793
Si30@FSA_6h - DNA@FSA_6h	0.930	Si10@FSA_12h - DNA-bSi10@FSA_12h	0.771
Si30@FSA_6h - DNA-bSi10@FSA_6h	0.884	Si10@FSA_12h - DNA-bSi20@FSA_12h	0.210
Si30@FSA_6h - DNA-bSi20@FSA_6h	0.307	Si10@FSA_12h - DNA-bSi30@FSA_12h	0.071
Si30@FSA_6h - DNA-bSi30@FSA_6h	0.031*	Si20@FSA_12h - Si30@FSA_12h	0.953
Si30@FSA_6h - FSA_12h	0.484	Si20@FSA_12h - DNA@FSA_12h	0.977
Si30@FSA_6h - Si10@FSA_12h	0.382	Si20@FSA_12h - DNA-bSi10@FSA_12h	1.000
Si30@FSA_6h - Si20@FSA_12h	0.243	Si20@FSA_12h - DNA-bSi20@FSA_12h	0.336
Si30@FSA_6h - Si30@FSA_12h	0.221	Si20@FSA_12h - DNA-bSi30@FSA_12h	0.129
Si30@FSA_6h - DNA@FSA_12h	0.255	Si30@FSA_12h - DNA@FSA_12h	0.930
Si30@FSA_6h - DNA-bSi10@FSA_12h	0.243	Si30@FSA_12h - DNA-bSi10@FSA_12h	0.953
Si30@FSA_6h - DNA-bSi20@FSA_12h	0.033*	Si30@FSA_12h - DNA-bSi20@FSA_12h	0.366
Si30@FSA_6h - DNA-bSi30@FSA_12h	0.007**	Si30@FSA_12h - DNA-bSi30@FSA_12h	0.145
DNA@FSA_6h - DNA-bSi10@FSA_6h	0.816	DNA@FSA_12h - DNA-bSi10@FSA_12h	0.977
DNA@FSA_6h - DNA-bSi20@FSA_6h	0.210	DNA@FSA_12h - DNA-bSi20@FSA_12h	0.321
DNA@FSA_6h - DNA-bSi30@FSA_6h	0.025*	DNA@FSA_12h - DNA-bSi30@FSA_12h	0.122
DNA@FSA_6h - FSA_12h	0.431	DNA-bSi10@FSA_12h - DNA-bSi20@FSA_12h	0.336
DNA@FSA_6h - Si10@FSA_12h	0.336	DNA-bSi10@FSA_12h - DNA-bSi30@FSA_12h	0.129
DNA@FSA_6h - Si20@FSA_12h	0.210	DNA-bSi20@FSA_12h - DNA-bSi30@FSA_12h	0.580

Table S9. Intergroup statistical comparisons of antioxidant ability and ROS scavenging effect of hydrogel.

Sample 1 - Sample 2	Significance	
	Antioxidant ability	ROS scavenging effect
Control - H_2O_2	0.000****	0.004***
Control - FSA	0.000****	0.048*
Control - DNA@FSA	0.000****	0.199
Control - Si30@FSA	0.000****	0.231
Control - DNA-bSi30@FSA	0.002***	0.701
H_2O_2 - FSA	0.225	0.716
H_2O_2 - DNA@FSA	0.005**	0.263
H_2O_2 - Si30@FSA	0.002***	0.228
H_2O_2 - DNA-bSi30@FSA	0.000****	0.050
FSA - DNA@FSA	0.264	0.943
FSA - Si30@FSA	0.120	0.914
FSA - DNA-bSi30@FSA	0.002***	0.426
DNA@FSA - Si30@FSA	0.994	1.000
DNA@FSA - DNA-bSi30@FSA	0.089	0.891
Si30@FSA - DNA-bSi30@FSA	0.201	0.925

Table S10. Intergroup statistical comparisons of relative dermis thickness, thickness of epidermis, thickness of granulation tissue, and collagen density of skin tissue extracted from diabetic mouse model after 15 days.

Sample 1 - Sample 2	Significance			
	Relative dermis thickness	Thickness of epidermis	Thickness of granulation tissue	Collagen density
Saline - FSA	0.715	Not statistically different between the groups	Not statistically different between the groups	0.768
Saline - DNA@FSA	0.144			0.168
Saline - Si30@FSA	0.028*			0.023*
Saline - DNA-bSi30@FSA	0.005**			0.001***
FSA - DNA@FSA	0.273			0.685
FSA - Si30@FSA	0.068			0.140
FSA - DNA-bSi30@FSA	0.014*			0.005**
DNA@FSA - Si30@FSA	0.465			0.699
DNA@FSA - DNA-bSi30@FSA	0.171			0.034*
Si30@FSA - DNA-bSi30@FSA	0.523			0.242

Table S11. Intergroup statistical comparisons of immunohistochemical factors (TGF-β, MPO, VEGF, and CD31) of skin tissue extracted from diabetic mouse model after 15 days.

Sample 1 - Sample 2	Significance			
	TGF-β	MPO	VEGF	CD31
Saline - FSA	0.523	0.523	0.300	0.535
Saline - DNA@FSA	0.121	0.055	0.110	0.049*
Saline - Si30@FSA	0.018*	0.045*	0.004***	0.003***
Saline - DNA-bSi30@FSA	0.001***	0.001***	0.003***	0.000****
FSA - DNA@FSA	0.361	0.201	0.952	0.468
FSA - Si30@FSA	0.083	0.171	0.102	0.028*
FSA - DNA-bSi30@FSA	0.011*	0.011*	0.071	0.000****
DNA@FSA - Si30@FSA	0.411	0.927	0.280	0.349
DNA@FSA - DNA-bSi30@FSA	0.100	0.201	0.202	0.001***
Si30@FSA - DNA-bSi30@FSA	0.411	0.235	0.999	0.013*

Table S12. Intergroup statistical comparisons of L929 proliferation by different DNA concentration.

Sample 1 - Sample 2	Significance		
	1d	3d	5d
0 μg/mL - 25 μg/mL	0.022*	0.014*	0.002***
0 μg/mL - 50 μg/mL	0.182	0.171	0.937
0 μg/mL - 100 μg/mL	0.004***	0.273	0.136
0 μg/mL - 500 μg/mL	0.917	0.411	0.000****
25 μg/mL - 50 μg/mL	0.650	0.273	0.007**
25 μg/mL - 100 μg/mL	0.768	0.171	0.000****
25 μg/mL - 500 μg/mL	0.079	0.001***	0.000****
50 μg/mL - 100 μg/mL	0.154	0.784	0.042*
50 μg/mL - 500 μg/mL	0.521	0.028*	0.000****
100 μg/mL - 500 μg/mL	0.013*	0.055	0.000****

Table S13. Intergroup statistical comparisons of hemolysis rates.

Sample 1 - Sample 2	Significance
Positive control - FSA	0.000****
Positive control - DNA@FSA	0.000****
Positive control - Si30@FSA	0.000****
Positive control - DNA-bSi30@FSA	0.000****
FSA - DNA@FSA	1.000
FSA - Si30@FSA	1.000
FSA - DNA-bSi30@FSA	1.000
DNA@FSA - Si30@FSA	1.000
DNA@FSA - DNA-bSi30@FSA	1.000
Si30@FSA - DNA-bSi30@FSA	1.000

Table S14. Intergroup statistical comparisons of relative dermis thickness, thickness of epidermis, thickness of granulation tissue, and collagen density of skin tissue extracted from acute wound healing model after 10 days.

Sample 1 - Sample 2	Significance			
	Relative dermis thickness	Thickness of epidermis	Thickness of granulation tissue	Collagen density
Saline - FSA	0.992	Not statistically different between the groups	0.315	0.768
Saline - DNA@FSA	0.524		0.144	0.168
Saline - Si30@FSA	0.043*		0.036*	0.023*
Saline - DNA-bSi30@FSA	0.012*		0.001***	0.001***
FSA - DNA@FSA	0.763		0.648	0.685
FSA - Si30@FSA	0.083		0.273	0.140
FSA - DNA-bSi30@FSA	0.023*		0.028*	0.005**
DNA@FSA - Si30@FSA	0.432		0.523	0.699
DNA@FSA - DNA-bSi30@FSA	0.141		0.083	0.034*
Si30@FSA - DNA-bSi30@FSA	0.916		0.273	0.242

Table S15. Intergroup statistical comparisons of immunohistochemical factors (TGF-β, MPO, VEGF, and CD31) of skin tissue extracted from acute wound healing model after 10 days.

Sample 1 - Sample 2	Significance			
	TGF-β	MPO	VEGF	CD31
Saline - FSA	0.528	0.273	Not statistically different between the groups	0.803
Saline - DNA@FSA	0.251	0.171		0.024*
Saline - Si30@FSA	0.001***	0.014*		0.002***
Saline - DNA-bSi30@FSA	0.000****	0.001***		0.000****
FSA - DNA@FSA	0.970	0.784		0.132
FSA - Si30@FSA	0.009**	0.171		0.009**
FSA - DNA-bSi30@FSA	0.001***	0.028*		0.001***
DNA@FSA - Si30@FSA	0.022*	0.273		0.437
DNA@FSA - DNA-bSi30@FSA	0.002***	0.055		0.037*
Si30@FSA - DNA-bSi30@FSA	0.490	0.411		0.471

Supporting movie file

Video S1. On-demand 3D printing of patient-customized functional hydrogel dressings with a non-lattice pattern (left) and a lattice pattern (right) using a diabetic ulcer foot model.
